# A pathological phosphorylation pattern enhances tau cooperativity on microtubules and facilitates tau filament assembly

**DOI:** 10.1038/s42003-026-10797-6

**Published:** 2026-08-21

**Authors:** Xiangyu Fan, Joey B. Truong, Kyoko Okada, Henry Lin, Kassandra M. Ori-McKenney, Richard J. McKenney

**Affiliations:** 1 School of Basic Medical Sciences, Henan University, Kaifeng, China; 2 Henan Provincial Engineering Centre for Tumor Molecular Medicine, Henan University, Kaifeng, China; 3 Department of Molecular and Cellular Biology, University of California, Davis, CA USA

**Keywords:** Biophysical chemistry, Molecular neuroscience

## Abstract

Phosphorylation of the microtubule-associated protein tau plays important roles in both normal physiology and disease. While tau phosphorylation levels are similarly elevated in processes such as fetal development and hibernation and in pathological states like Alzheimer’s disease, the pattern of phosphorylation differs between these contexts. Whether distinct phosphorylation patterns differentially impact tau’s function on microtubules and its propensity to form neurofibrillary tangles (NFTs) has remained unclear. Here we investigated how differential phosphorylation affects microtubule binding, cooperative tau envelope formation, and NFT-relevant filament assembly. Consistent with prior work, phosphorylation decreases tau’s overall microtubule affinity. However, we find that phosphorylation more dramatically impacts cooperativity between tau molecules during envelope formation. Strikingly, the specific pattern of phosphorylation, rather than overall level, strongly promotes tau filament assembly in vitro. These findings provide insights into how tau phosphorylation modulates its physiological microtubule interactions and its pathoconversion into NFTs.

**Figure:**
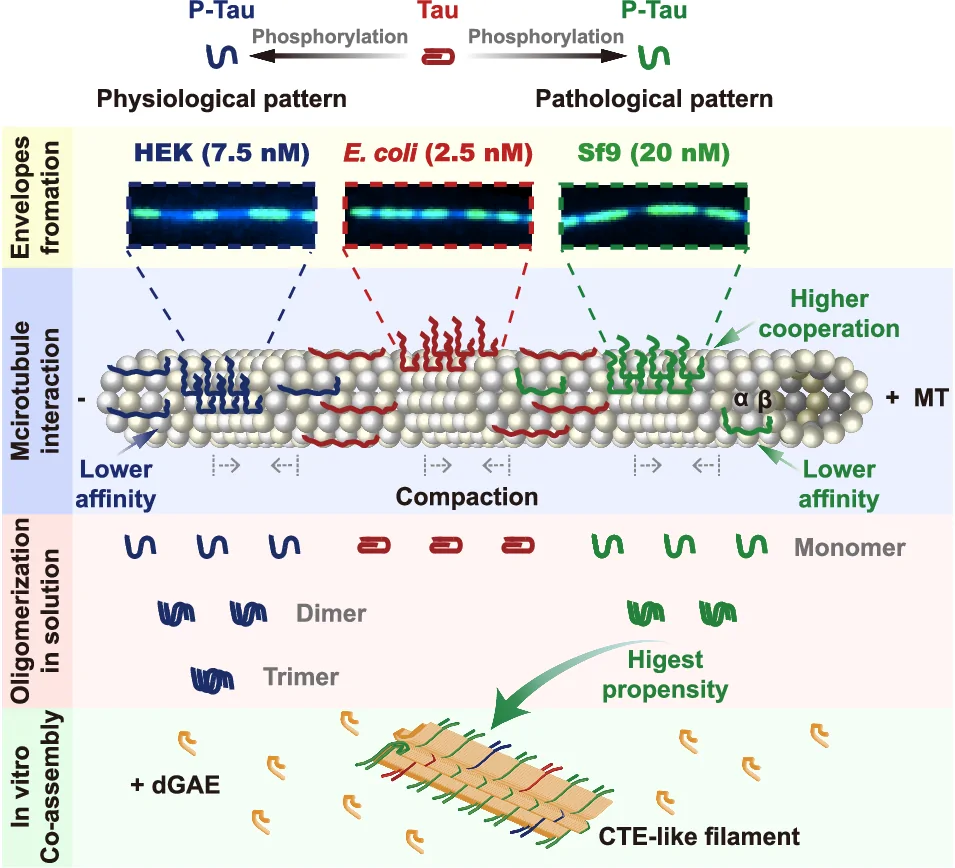

## Abstract (web summary)

Pathological-like tau phosphorylation enhances tau cooperativity on microtubules and accelerates co-assembly into CTE-like fibrils, showing that phosphorylation pattern rather than level drives key disease-relevant behaviors.

## Introduction

Tau was first identified as a microtubule-associated protein (MAP) in 1975^1^, and was initially thought to play a key role in regulating microtubule dynamics by promoting polymerization and stability^2,3^. Further research revealed that tau also participates in many cellular processes, including the activation of MAPK (mitogen-activated protein kinase)^4^, the regulation of synaptic function^5–7^, the protection of RNA and DNA in nucleus^8–11^, and the regulation of protein synthesis^12^. More recently, we and others have shown that tau plays a crucial role in regulating microtubule-based activities by forming cooperative protein clusters termed “envelopes” along microtubules, which act as selectively permeable barriers for other MAPs^13,14^, impacting microtubule-based cargo transport^15,16^. These envelopes arise when tau molecules transition from discrete single-molecule binding to a continuous, sheath-like coating along the microtubule surface, driven by cooperative interactions between adjacent, microtubule-bound tau molecules^13,14^. In this process, these cohesive tau envelopes work as a microtubule remodeler to compact the lattice of the microtubule^17,18^.

Research interest in tau rose sharply after 1985 when tau was identified as a primary constituent of the neurofibrillary tangles (NFTs) in Alzheimer’s disease (AD) patient’s brain^19–22^, as well as other neurodegenerative diseases, such as chronic traumatic encephalopathy (CTE)^23,24^, progressive supranuclear palsy (PSP) and Pick’s disease (PiD)^25^. NFTs are composed of paired-helical filaments (PHFs) and straight filaments (SFs), which are polymers of tau protein that have undergone a structural transition from an intrinsically disordered state to a highly ordered cross-β structure. Recent high-resolution structural studies have elucidated the atomic arrangement of these tau filaments, providing insights into this structural transformation^26–29^. However, the molecular mechanism underlying the conversion of tau protein into this highly ordered state remains poorly understood and is the subject of ongoing research. The accumulation of NFTs in tauopathies is closely linked to neuronal cell death and resulting cognitive decline, ultimately leading to dementia and death. The link between tau’s physiological roles and conversion to pathological states remains undefined and stands as one of the great mysteries obfuscating a fuller understanding of the dementia process.

Phosphorylation of tau plays a key role in mediating tau’s physiological and pathological roles. Tau phosphorylation is a hallmark of NFTs in various tauopathies^20–25^. Research has consistently shown that multi-site phosphorylation of tau alters its conformation, reduces its affinity for microtubules, and promotes aggregation^30–39^. Additionally, abnormal tau phosphorylation leads to mislocalization of tau from the axon to the cytosol within neurons^40^. Interestingly, phosphorylation also plays a crucial role in normal physiological processes, such as animal hibernation^41–43^, and fetal development^44–46^. In hibernating animals, tau protein exhibits elevated phosphorylation levels during torpor, a state characterized by reduced metabolic rate and body temperature. This phenomenon is likely influenced by the altered activity of kinases and phosphatases, which are sensitive to the low metabolic and thermal conditions^43^. Following arousal from hibernation, the phosphorylation levels of tau protein return to normal. Notably, this hibernation-arousal cycle does not result in brain damage, suggesting that the brain is resilient to changes in tau protein phosphorylation that occur during torpor^41–43,47^. In fetal brain development, tau is highly phosphorylated at multiple sites, but this phosphorylation level returns to a steady state after maturation due to increased phosphatase activity in adult brains^44^. Interestingly, fetal tau shows a similar phosphorylation level to the tau from AD brains, but with no signs of aggregation or NFT formation^39,45,46,48^. Furthermore, physiological tau phosphorylation is typically thought to be a reversible process, unlike pathological tau phosphorylation^41–43^.

The physiological functions of phosphorylated tau, including its regulation of microtubule-based activities, remain poorly understood. Furthermore, the impact of phosphorylation on the interactions between tau molecules on microtubules and on the assembly of pathological NFTs remains unclear. To address these knowledge gaps, we employed tau proteins with varying phosphorylation patterns and investigated the effects of phosphorylation on microtubule binding, cooperative tau envelope formation, oligomerization, and filament assembly, all of which are relevant to tauopathies. Our findings reveal that tau phosphorylation alters the single-molecule kinetics and cooperative interactions that underlie tau envelope formation along microtubules. However, phosphorylated tau can still bind to and compact the microtubule lattice, albeit with reduced affinity. We discovered that human tau protein produced in insect cells, which exhibits a phosphorylation pattern similar to that found in Alzheimer’s disease brains^49^, alters cooperative interactions between tau molecules and impacts cooperative tau envelope assembly. Additionally, our results show that phosphorylation promotes oligomerization of full-length tau molecules in solution. Finally, in vitro co-assembly assays revealed that pathological phosphorylation patterns greatly facilitate the assembly of tau proteins into NFT-like filaments under conditions that promote filament structures found in CTE^50,51^. Our findings suggest that pathological phosphorylation patterns, but not phosphorylation generally, not only alter cooperation between tau molecules during envelope formation along microtubules but also promote the assembly of filaments relevant to tauopathies.

## Results

### Characterization of differentially phosphorylated recombinant human tau proteins

**Fig. 1:**
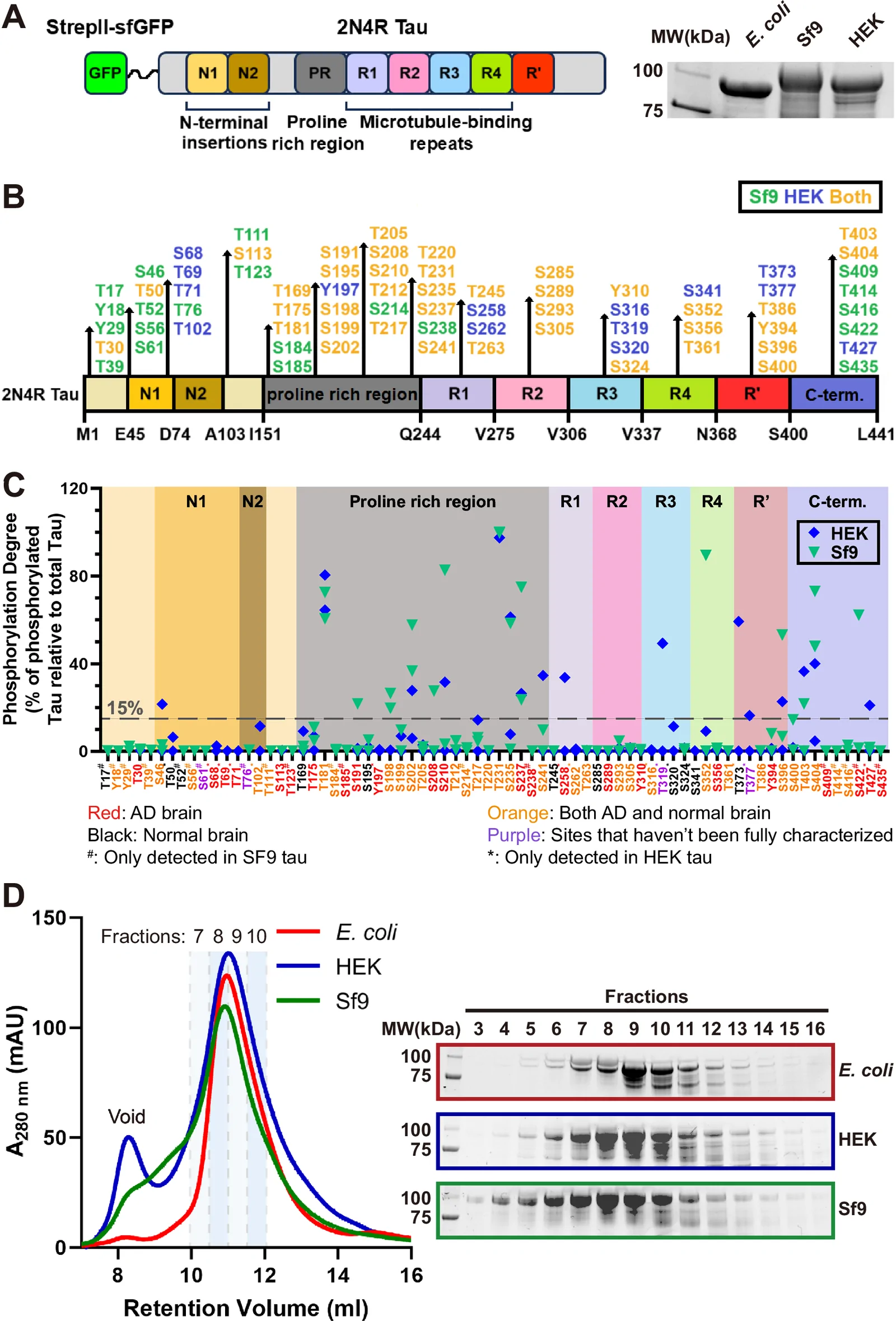
Characterization of differentially phosphorylated tau proteins. **A** Schematic of the human 2N4R tau construct used in this study, which contains N-terminal N1 and N2 insertions, a proline-rich region, 4 microtubule binding repeats and a C-terminal pseudo-repeat region (R’). A Coomassie-stained SDS-PAGE gel showing the differentially phosphorylated human 2N4R tau proteins, which were expressed and purified from different expression systems, is shown. Note: the precise definition of the microtubule binding repeats has not always been consistent in previous literature, we defined the microtubule binding repeats as residues 242–367, similar to a previous cryo-EM study, in which the R’ is not included^70^. **B** Schematic showing the distribution of the phosphorylation sites in HEK tau and Sf9 tau detected by mass spectrometry. Sites only detected in HEK tau are labeled dark blue, sites only detected in Sf9 tau are labeled dark green, and sites detected in both HEK tau and Sf9 tau are labeled amber/yellow. **C** Schematic showing the site-specific phosphorylation degrees at corresponding sites in the HEK tau (dark blue) and the Sf9 (dark green) tau based on the calculation from mass spectrometry. Two independent experiments (*n* = 2) were performed. Annotation shows the phosphorylation sites detected in either AD or normal brain (summarized from previous studies^59–65^). **D** Size exclusion chromatograph of differentially phosphorylated tau proteins preparations. A SDS-PAGE gel of peak fractions shows only subtle differences between tau proteins purified from different expression systems. A tag-cleaved SEC control for *E. coli* and Sf9 tau is shown in Supplementary Fig. 1.

To generate differentially phosphorylated tau proteins, we expressed the longest isoform of human tau (441 amino acids, 2N4R) in bacteria (*E. coli*), insect cells (*Spodoptera frugiperda* 9 cells, Sf9) and mammalian cells (Human Embryonic Kidney Cells 293, HEK293), referred to as *E. coli* tau, Sf9 tau and HEK tau, respectively, hereafter. All proteins were purified via a N-terminal StrepII-superfolder GFP (sfGFP) tag and were analyzed by sodium dodecyl sulfate-polyacrylamide gel electrophoresis (SDS-PAGE). Both Sf9 tau and HEK tau exhibited a slower migration rate compared with their non-phosphorylated cognate *E. coli* tau (Fig. 1A), presumably due to the alteration of protein molecular weight or charge introduced by phosphorylation. The 2N4R tau protein has 85 potential phosphorylation sites, consisting of 45 serines, 35 threonines, and 5 tyrosines^3,52,53^. To identify the phosphorylated residues in our tau preparations, we used liquid chromatography-electrospray mass spectrometry (LC-MS). Our analysis revealed that the HEK tau protein contained 52 phosphorylated sites, while the Sf9 tau protein contained 58 phosphorylated sites. Both protein preparations share 38 common phosphorylation sites (Fig. 1B). We conclude that human tau protein expressed in heterologous eukaryotic cells is differentially phosphorylated by endogenous cellular kinases.

To quantify the phosphorylation status of these tau proteins, we determined the site-specific phosphorylation levels of both HEK tau and Sf9 tau by comparing the signal intensity of phosphorylated peptides at specific sites to the total signal intensity of both phosphorylated and non-phosphorylated peptides within the same site^54,55^. This analysis revealed that the phosphorylation occupancy in most sites of both HEK tau and Sf9 tau was lower than ~15% (Supplementary Table 1). Most sites where the phosphorylation occupancy was higher than 15% were located outside of the microtubule binding repeats (Fig. 1C). Specifically, in HEK tau, seven sites located in the proline rich region (T181, S202, S210, T231, S235, S237 and S241), three sites located in the C-terminus (T373, T403 and S404) and 2 sites (S258 and T319) located in the microtubule binding repeat had an occupancy of 15% or higher (Fig. 1C and Supplementary Table 1). In the Sf9 tau protein, we identified 11 sites with phosphorylation occupancy greater than 15%. These sites are distributed across three regions: the proline-rich region (seven sites: T181, S198, S202, S210, T231, S235, and S237), the C-terminal region (three sites: S396, S404, and S422), and the microtubule binding repeat (1 site: S352, Fig. 1C and Supplementary Table 1). The distribution of phosphorylated sites in our Sf9 tau is similar to a previous report^54,56^. These results reveal that specific sites within the tau molecule are more susceptible to phosphorylation by endogenous kinases.

Among the highly phosphorylated sites, T181 and T231 are phosphorylated in both HEK tau and Sf9 tau, and have been previously reported as key sites in Alzheimer’s disease (AD)^57^. Phosphorylated T181 and T231 have been identified as biomarkers for AD^58^, and previous studies have shown that they are highly phosphorylated in Sf9 tau as well^49,56^. Additionally, phosphorylation at S241, S258, T319, and T373 is specific to HEK tau or is significantly higher in HEK tau than in Sf9 tau. Conversely, phosphorylation at S198, S202, S210, S237, S352, S396, S404 and S422 is specific to Sf9 tau or is significantly higher in Sf9 tau than in HEK tau (Fig. 1C and Supplementary Table 1). Notably, the phosphorylation pattern in Sf9 tau includes sites such as S210, S237, and S422, which are prominently featured within the complex phosphoproteome of AD brains and represent a pathologically relevant constellation of modifications (Fig. 1C)^59–65^.

Previous research has shown that healthy brains typically contain 2–3 phosphates per tau molecule. In NFTs, however, this number is aberrantly increased to 5-9^66,67^. Our findings are consistent with these reports, as we observed an average phosphorylation stoichiometry of ~5 phosphates per molecule in HEK tau and ~6 phosphates per molecule in Sf9 tau (Supplementary Table 1). This was determined by quantifying and summing the fractional occupancy for all detected phosphosites. Because HEK tau and Sf9 tau are heterogeneous ensembles of phospho-isoforms, the present study tests the functional consequences of phosphorylation patterns at the population level rather than assigning specific effects to individual phospho-isoforms. We note that previous studies have used the term “hyperphosphorylation” to describe insect cell-expressed tau. However, given that the overall level of phosphorylation is similar between our two preparations, we will use more specific terms such as “physiological-like” (HEK) and “pathological-like” (Sf9) phosphorylation to emphasize that the phosphate pattern, not the overall amount, is the key variable in this study. These results demonstrate that while HEK tau and Sf9 tau share similar levels of phosphorylation, the phosphorylation patterns in these two proteins are distinct.

Given the distinct phosphorylation patterns observed in HEK tau and Sf9 tau, we sought to investigate whether these differences impact the conformation or ensemble oligomeric state of tau molecules in solution. To address these questions, we first employed size exclusion chromatography to analyze the apparent molecular size of both HEK tau and Sf9 tau, using non-phosphorylated *E. coli* tau as a reference control. The chromatographic profiles revealed that HEK tau eluted at a highly similar elution volume to *E. coli* tau, whereas Sf9 tau eluted slightly earlier than both *E. coli* tau and HEK tau (Fig. 1D). This subtle shift in Sf9 tau could suggest a modest conformational change in this phosphorylation variant. To assess whether the large N-terminal sfGFP tag might contribute to this result, we repeated the SEC analysis with *E. coli* tau and Sf9 tau following proteolytic removal of the tag (Supplementary Fig. 1). The earlier elution of tag-free Sf9 tau relative to tag-free *E. coli* tau was preserved, confirming that this shift reflects a property of the tau molecule itself rather than an artifact of the sfGFP fusion. We were unable to generate sufficient quantities of tag-free HEK tau for this comparison, and therefore interpret the HEK tau elution profile with the caveat that a potential tag contribution cannot be formally excluded. Nonetheless, all three preparations eluted as largely homogenous populations under these conditions, and more definitive evidence for functional differences between these variants is provided by subsequent assays.

Taken together, these findings demonstrate that we have produced differentially phosphorylated tau proteins that exhibit a similar distribution of phosphorylation sites but with distinct phosphorylation patterns. Further, phosphorylation of tau does not dramatically alter its ensemble oligomeric state or hydrodynamic radius.

### Effects of phosphorylation on single-molecule and ensemble microtubule binding behaviors of tau

**Fig. 2:**
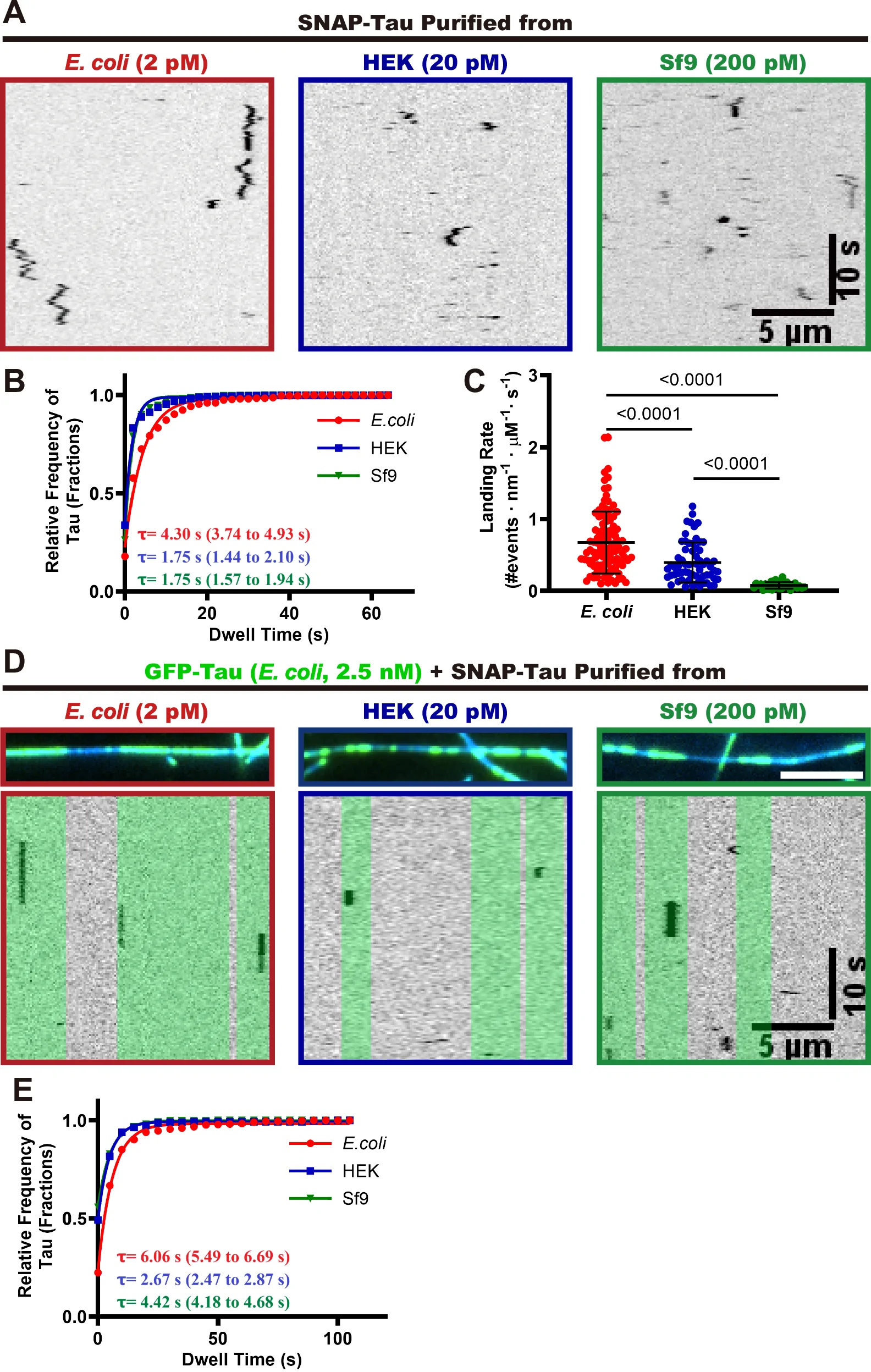
Single-molecule analysis of the effects of phosphorylation on the tau-microtubule interaction. **A** Kymographs of differentially phosphorylated single SNAP-tau molecules diffusing on bare microtubules. Scale bars: 10 s. and 5 µm. **B** Quantification of the dwell times of differentially phosphorylated tau proteins on microtubules. Cumulative frequency of the dwell time is plotted for each population of tau proteins and fit to a one-phase exponential decay function. The characteristic dwell times derived from the fits (τ) and the corresponding 95% confidence intervals (95% CI, in parentheses) are indicated. Events longer than three pixels were selected and quantified and two independent experiments (*n* = 2) were performed for each condition. *E. coli* tau: *N* = 330, R^2^ = 0.978. HEK tau: *N* = 831, R^2^ = 0.973. Sf9 tau: *N* = 726, R^2^ = 0.990. **C** Quantification of landing rates of differentially phosphorylated tau proteins on microtubules. For quantification, microtubules were quantified from two independent experiments (*n* = 2) for each tau protein. *E. coli* tau: *N* = 106 microtubules quantified. HEK tau: *N* = 63. Sf9 tau: *N* = 31 microtubules quantified. *P* values are calculated from an unpaired, two-tailed *t*-test. Mean ± SD is shown. **D** Kymographs of differentially phosphorylated SNAP-tau proteins within non-phosphorylated GFP-tau envelopes on microtubules. Tau envelope boundaries are colored green on the kymographs. Scale bars: 10 s. and 5 µm. **E** Quantification of the dwell times of differentially phosphorylated SNAP-tau proteins within envelopes formed by non-phosphorylated GFP-tau. Cumulative frequency of dwell time is plotted for each tau protein population and fit to a one-phase exponential decay function. The characteristic dwell times derived from the fits (τ) and the corresponding 95% CI (in parentheses) are indicated. Events longer than three pixels were selected and quantified and two independent experiments were performed for each condition. *E. coli* tau: *N* = 430, R^2^ = 0.993. HEK tau: *N* = 352, R^2^ = 0.998. Sf9 tau: *N* = 353, R^2^ = 0.998. For each tau protein, two independent experiments (*n* = 2) were performed.

Tau phosphorylation may influence its interaction with microtubules by altering the protein’s charge status and/or conformation, which could disrupt the formation of salt bridges between tau and microtubules^30,33,53,68–70^. To investigate whether phosphorylation affects the dynamics of tau on microtubules, we first examined the behavior of differentially phosphorylated tau proteins at picomolar concentrations, enabling single-molecule observation. We tracked the binding dynamics of single SNAP-tagged 2N4R tau molecules along surface-immobilized taxol-stabilized microtubules (Fig. 2A). We quantified the single molecule dwell times and landing rates, revealing that phosphorylated tau molecules displayed an ~ 2.5-fold shorter dwell time on microtubules (*E. coli* tau: ~ 4.3 s, HEK and Sf9 tau: ~ 1.75 ss, Fig. 2B). Additionally, the landing rates of phosphorylated tau molecules were reduced by ~ 2-10-fold (*E. coli* tau: 0.67 ± 0.43 nM^-1^μm^-1^s^-1^, HEK tau: 0.40 ± 0.28 nM^-1^μm^-1^s^-1^, Sf9 tau: 0.08 ± 0.05 nM^-1^μm^-1^s^-1^, Fig. 2C). To directly confirm that these differences are driven by phosphorylation rather than other post-translational modifications present on eukaryotic cell-expressed tau, we treated Sf9 tau with Lambda Protein Phosphatase (LPP). Dephosphorylation significantly increased both the dwell time ( ~ 1.59 s to ~4.42 s) and the landing rate of Sf9 tau on microtubules (Supplementary Fig. 2A–D), restoring values to a range comparable to those of non-phosphorylated *E. coli* tau (Fig. 2B, C). These results confirm that phosphorylation is the primary determinant of the reduced microtubule-binding dynamics observed for Sf9 tau. Overall, our results show that tau phosphorylation decreases the initial binding rate and dwell time of tau with microtubules, consistent with previous bulk biochemical studies that showed the tau phosphorylation lowers its overall microtubule affinity^30–33,56^.

We and others have proposed that one of the key functions of the tau protein is its ability to form cohesive tau envelopes on the microtubule surface in a concentration-dependent manner^13,14,17,18,56^. These tau envelopes act as a selectively permeable barrier to regulate the activities of other microtubule-associated proteins (MAPs)^13,14^. Within the envelope, tau proteins exhibit cooperative interactions with each other, resulting in longer dwell times compared to tau proteins outside the envelope^13,14^. To investigate whether phosphorylation affects the interaction of tau proteins within these envelopes, we employed a single-molecule assay to measure the dwell times of differentially phosphorylated tau proteins within envelopes formed by non-phosphorylated *E. coli* tau. In this assay, 2.5 nM of GFP-tagged *E. coli* tau was preincubated with taxol-stabilized microtubules to form envelopes, followed by the introduction of differentially phosphorylated and SNAP-labeled tau proteins. This concentration (2.5 nM) was chosen as a sub-saturating condition sufficient to form clear *E. coli* tau envelopes to probe the cooperative interactions of the subsequently added phosphorylated tau species. Our results show that all three tau proteins can engage with the envelopes formed by non-phosphorylated *E. coli* tau (Fig. 2D).

We next examined the dwell times of the tau proteins within the *E. coli* tau envelopes. The measured dwell time of single *E. coli* tau molecules was ~ 6.06 s, while the dwell times of phosphorylated HEK tau and Sf9 tau molecules were reduced to ~2.7 s and ~4.4 s, respectively (Fig. 2D, E). Notably, all three tau proteins exhibited increased dwell times within the envelopes compared to their dwell times outside of the envelopes (Fig. 2B), confirming the existence of cooperative interactions between the differentially phosphorylated tau proteins within the envelopes. The shorter dwell times of phosphorylated tau molecules within tau envelopes suggest that phosphorylation impairs the cooperative interaction between phosphorylated and non-phosphorylated tau proteins within the envelopes, consistent with a previous study, which found that Cdk5 phosphorylation can induce disassembly of envelopes formed by non-phosphorylated bacterial tau^56^. Interestingly, we found that HEK tau exhibited the shortest dwell times within the envelopes, ~ 2.3 fold shorter than *E. coli* tau. We speculate that this is because phosphorylation at T373, and to a lesser extent T377, both of which lie within the pseudo-repeat region R’ and are HEK-specific modifications, interferes with the tau-tau cooperative interactions that R’ is known to mediate^13^ (Fig. 1C and Supplementary Table 1). T373 in particular, phosphorylated at ~30% occupancy, would introduce a negatively charged phosphate group directly into this critical domain, potentially disrupting favorable tau-tau contacts via electrostatic repulsion or steric hindrance. Our results show that phosphorylation impairs the cooperative interaction between tau molecules within envelopes formed by non-phosphorylated tau, and that specific phosphorylation sites may differentially impact these interactions that underlie tau envelope formation.

### Phosphorylated tau retains the capability to form envelopes and to compact the microtubule lattice

**Fig. 3:**
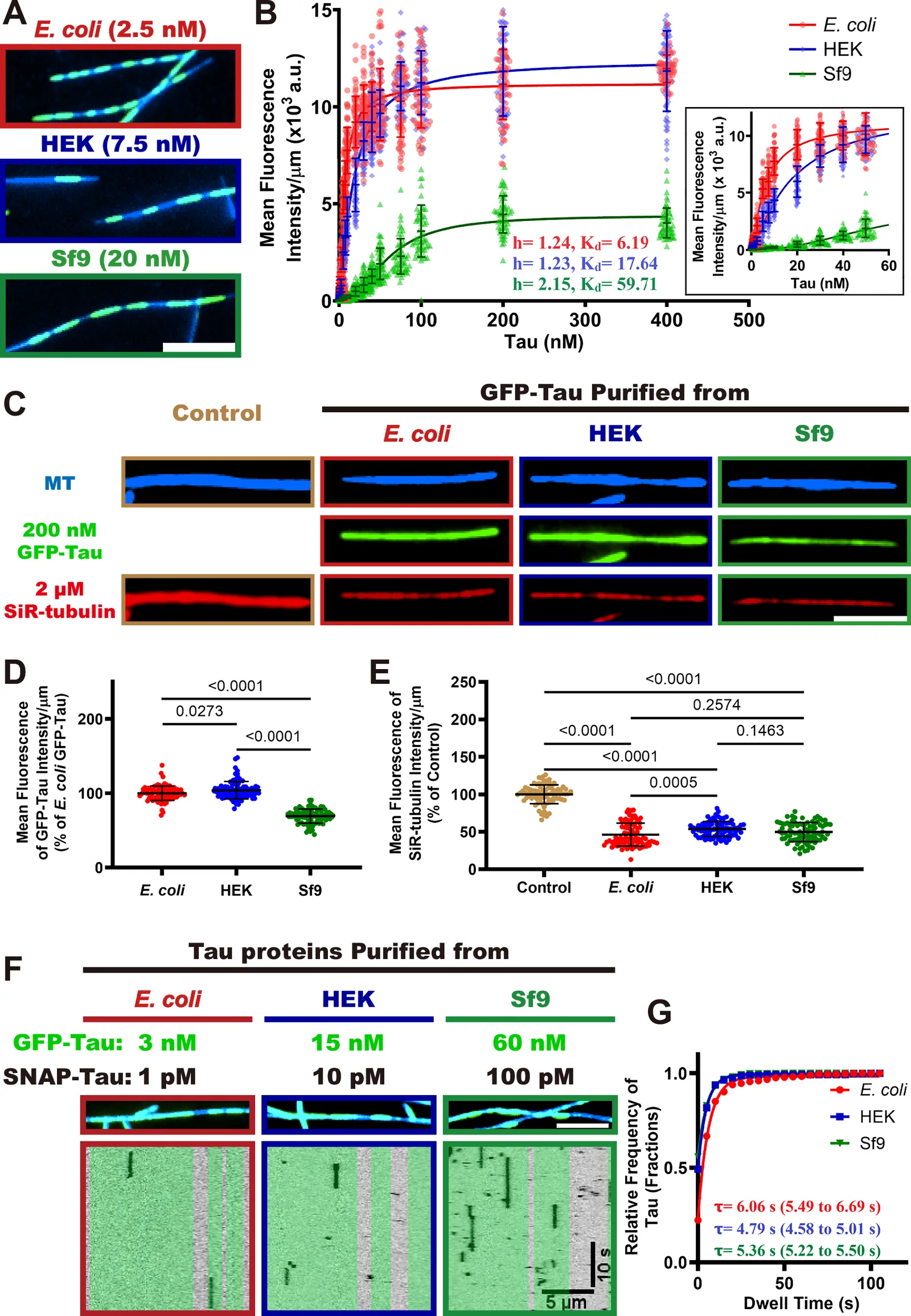
Specific phosphorylation patterns alter cooperative interactions between tau proteins on microtubules. **A** TIRF images showing GFP-tau envelopes on taxol-stabilized microtubules. Scale bar: 5 µm. **B** Quantification of differentially phosphorylated tau proteins binding to microtubules across a range of tau concentrations. Binding curves were fitted to the Hill equation to derive the Hill coefficient and *K*_*d*_. The *K*_*d*_ reflects the overall binding affinity, while the Hill coefficient quantifies the degree of positive cooperativity (h > 1). Each reported value is derived from a fit to a full binding curve generated from the titration series. For each concentration of the corresponding tau protein, two independent experiments (*n* = 2) were performed. *N* = 60 microtubules. Mean ± SD is shown. Hill coefficients and *K*_*d*_ are indicated. 95% CI for Hill coefficients: 1.16 to 1.33 (*E.coli* tau), 1.17 to 1.30 (HEK tau), 1.97 to 2.34 (Sf9 tau). 95% CI for *K*_*d*_: 5.84 to 6.60 (*E.coli* tau), 16.66 to 18.70 (HEK tau), 56.93 to 62.75 (Sf9 tau). Insert showing the initial time course of binding. The reported *K*_*d*_ and Hill coefficient for each tau variant were derived by fitting a full binding curve from a titration series of tau concentrations. **C** TIRF images showing microtubules and differentially phosphorylated tau proteins at saturating concentration (200 nM), along with SiR-tubulin bound along the microtubules. Scale bar: 5 µm. **D** Quantification of the average fluorescence intensities of differentially phosphorylated GFP-tau proteins on the microtubules. For quantification, GFP-tau were quantified for each condition from three independent experiments (*n* = 3). *E. coli* tau: *N* = 90. HEK tau: *N* = 90. Sf9 tau: *N* = 90. *P* values are calculated from an unpaired, two-tailed *t*-test. Mean ± SD is shown. **E** Quantification of the average fluorescence intensities of SiR-tubulin along the microtubules. For quantification, microtubules were quantified for each condition from three independent experiments (*n* = 3). Control condition: *N* = 90 microtubules. *E. coli*: *N* = 90. HEK tau: *n* = 90. Sf9 tau: *N* = 90. *P* values are calculated from an unpaired, two-tailed *t*test. Mean ± SD is shown. **F** Top panels showing envelopes formed by differentially phosphorylated GFP-tau proteins. Scale bar: 5 µm. Bottom panels showing kymographs of differentially phosphorylated SNAP-tau proteins within envelopes formed by corresponding GFP-tau proteins on the microtubules. Tau envelope boundaries are colored green on the kymographs. Scale bars: 10 s and 5 µm. **G** Quantification of the dwell times of differentially phosphorylated tau proteins within envelopes on the microtubules. Cumulative frequency of dwell time is plotted for each tau protein population and fit to a one-phase exponential decay function. The characteristic dwell times derived from the fits (τ) and the corresponding 95% CI (in parentheses) are indicated (the *E. coli* tau data were re-plotted from Fig. 2E for comparison). Events longer than three pixels were selected and quantified and two independent experiments (n = 2) were performed for each condition. *E. coli* tau: *N* = 413, R^2^ = 0.993. HEK tau: *N* = 311, R^2^ = 0.999. Sf9 tau: *N* = 430, R^2^ = 0.9995.

We have shown that phosphorylation reduces the affinity of single tau molecules for microtubules and diminishes the cooperative interactions within envelopes formed by non-phosphorylated tau. We next sought to investigate whether phosphorylation hinders tau’s ability to form envelopes. To address this question, we examined the capacity of differentially phosphorylated tau proteins to form envelopes on microtubules at various concentrations. Our results show that all three tau proteins retain the ability to form envelopes on microtubules, although Sf9 tau and HEK tau require substantially higher concentrations to do so (Fig. 3A). This observation is consistent with previous reports that higher concentrations of Sf9 tau are required to form envelopes on microtubules^13,56^. These findings indicate that while phosphorylation diminishes tau’s microtubule affinity and impairs the cooperative interactions within envelopes formed by non-phosphorylated tau, it does not fully eliminate its ability to form envelopes on microtubules.

Because tau envelope formation is a cooperative process^17^, we next sought to quantify how phosphorylation impacts the underlying molecular interactions. In this context, cooperative binding refers to the phenomenon where favorable lateral interactions between adjacent, MT-bound tau molecules facilitate the binding of additional tau. To measure this, we performed a microtubule binding assay across a range of tau concentrations and fit the resulting binding curves to the Hill equation (Fig. 3B). This analysis yields two key parameters: the apparent dissociation constant (K_d_), which reflects the overall tau-microtubule binding affinity, and the Hill coefficient (h), which quantifies the degree of cooperativity. We measured very similar Hill coefficients of 1.24 (95% Confidence Intervals, 1.16 to 1.33) for *E. coli* tau, and 1.23 (95% CI, 1.17 to 1.30) for HEK tau, indicating that the phosphorylation pattern found on HEK tau does not dramatically alter the cooperative interaction between HEK tau molecules during envelope formation (Fig. 3B). In contrast, the Hill coefficient of Sf9 tau was elevated to 2.15 (95% CI, 1.97 to 2.34), suggesting that this phosphorylation pattern, or phosphorylation of specific sites, enhances the cooperative interaction between Sf9 tau molecules. While our findings agree with the general consensus in the field that phosphorylation reduces microtubule affinity, our results on cooperativity diverge from recent work^56^, which reported a general reduction in the Hill coefficient following phosphorylation. The unique enhancement of cooperativity seen with the pathological-like Sf9 tau (h = 2.15) compared to the physiological-like HEK tau (h = 1.23) suggests that the specific pattern of phosphorylation is the key determinant of its effect on tau-tau cooperative interactions, a finding that builds upon and refines the current understanding of phosphorylation’s role in envelope stability.

To directly confirm that the enhanced cooperativity and reduced microtubule affinity of Sf9 tau are driven by phosphorylation rather than other post-translational modifications, we measured binding curves for LPP-treated Sf9 tau (Supplementary Fig. 2E). The LPP experiment was performed on an independent Sf9 tau preparation, which exhibited a Hill coefficient of 2.88 (95% CI, 2.54 to 3.32) and a K_d_ of ~ 54 nM, values within the range of batch-to-batch variability we observe across independent purifications of this protein. Dephosphorylation of this preparation reduced the Hill coefficient to 1.95 (95% CI, 1.80 to 2.12) and substantially increased microtubule affinity, decreasing the apparent K_d_ to ~ 10 nM. While these values do not fully recapitulate those of non-phosphorylated *E. coli* tau (h = 1.24, K_d_ = 6.19 nM), they move substantially in that direction, consistent with either incomplete dephosphorylation by LPP or a residual contribution from other post-translational modifications present on Sf9-expressed tau. Together with the single-molecule data in Supplementary Fig. 2A–D, these results confirm that phosphorylation is the primary determinant of the enhanced cooperativity and reduced microtubule affinity observed for Sf9 tau.

Furthermore, the dissociation constant (K_d_) values of *E. coli* tau, HEK tau, and Sf9 tau were 6.19 nM, 17.64 nM, and 59.71 nM, respectively (Fig. 3B), confirming that phosphorylation decreases the overall microtubule affinity of tau protein, consistent with our single-molecule results (Fig. 2). These findings collectively demonstrate that phosphorylation does not strongly disrupt, and can even enhance, the cooperative interaction between tau proteins within envelopes, while also reducing their overall microtubule affinity. Notably, the maximum binding capacity (B_max_) of Sf9 tau was ~ 3-fold lower than that of *E. coli* and HEK tau (Fig. 3B), which may be attributed to the lower landing rate of Sf9 tau (Fig. 2A–C). We speculate that Sf9 phosphorylation may also induce a conformational change, as supported by our gel filtration results (Fig. 1D), which may conceivably affect envelope formation and density. Further investigation is needed to confirm this hypothesis.

We have proposed that envelopes play a crucial role in regulating the accessibility of microtubule-associated proteins (MAPs) to the microtubule lattice^13,14^. One mechanism for this activity may be through compaction of the underlying tubulin lattice^17,18^. Given that the B_max_ of Sf9 tau is significantly lower than that of *E. coli* tau and HEK tau, we investigated whether Sf9 tau was competent to induce microtubule lattice compaction. We used a SiR-tubulin assay, in which the fluorescent docetaxel analog SiR-tubulin reports on microtubule lattice compaction in the presence of MAP proteins^17^, to assess whether differentially phosphorylated GFP-tau proteins can compact microtubule lattices. The assay was performed on taxol-stabilized microtubules with expanded lattices, preincubated with 2 μM SiR-tubulin on a coverslip^17^. We then added the phosphorylated tau proteins at saturating concentrations (200 nM) and measured the fluorescence intensities of both GFP-tau and SiR-tubulin using TIRF microscopy. Our results show that the saturating fluorescence intensity of Sf9 tau is approximately 69% lower than *E. coli* tau, while HEK tau was very similar to *E. coli* tau (Fig. 3C, D). This is consistent with the binding curve data obtained at saturating concentrations (Fig. 3B). The addition of *E. coli* tau to the microtubules resulted in a significant decrease in SiR-tubulin fluorescence intensity by ~ 50% (Fig. 3C, E), indicating that *E. coli* tau compacts the microtubule lattice and competes with SiR-tubulin for binding, consistent with prior results^17^. Interestingly, despite its 3-fold lower Bmax, Sf9 tau displaced SiR-tubulin to a similar degree as *E. coli* and HEK tau at 200 nM (Fig. 3E). This result suggests that microtubule lattice compaction does not scale linearly with tau-binding density but rather occurs once a minimum occupancy threshold is exceeded. Put differently, each tau molecule that successfully occupies a lattice binding site appears to induce an equivalent degree of tubulin compaction regardless of its phosphorylation state. That is, phosphorylation alters how many tau molecules ultimately bind, but not the structural change each bound molecule produces in the lattice. Consistent with this threshold model, the degree of Sf9 tau binding achieved at 200 nM, well above its K_d_ of ~60 nM, is sufficient to surpass the compaction threshold despite the lower absolute binding density.

We next measured the dwell times of single tau molecules within envelopes formed from the same type of tau preparation (non-phosphorylated or phosphorylated). Non-phosphorylated *E. coli* tau showed the longest dwell times, while HEK cell tau molecules showed somewhat extended dwell times inside phosphorylated HEK tau envelopes as compared to within dephosphorylated *E. coli* tau envelopes (Figs. 2D, E, 3F, G). This result indicates increased interactions among phosphorylated tau molecules within envelopes in HEK cells. Phosphorylated Sf9 cell tau molecules also showed modestly enhanced dwell times within Sf9 cell tau envelopes as compared to within *E. coli* tau envelopes (Figs. 2D, E, 3F, G). These results further confirm our observations that tau phosphorylation, while reducing overall microtubule affinity, also impacts tau’s cooperative interactions (Fig. 2B). We conclude that phosphorylated tau retains the capability to form cooperative envelopes and compact the microtubule lattice, which in turn regulates the microtubule-based activities, consistent with recent results showing that phosphorylated tau partially retains its function, which works as a selectively permeable barrier^14,56^.

### Phosphorylated tau forms small oligomers in solution

**Fig. 4:**
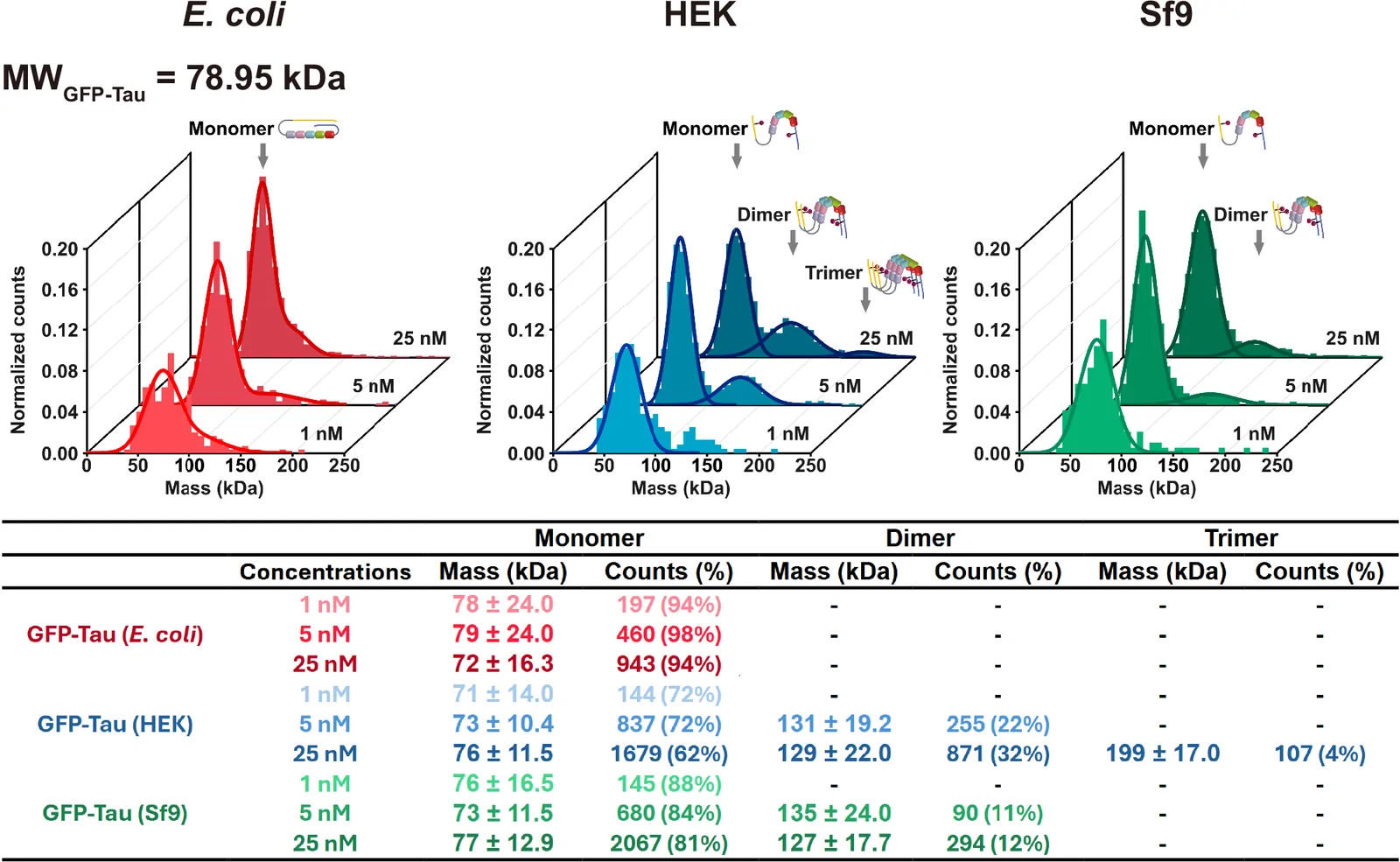
Phosphorylation promotes oligomerization of tau in solution. Characterization of the oligomeric state of differentially phosphorylated tau proteins by mass photometry. Histograms in each panel show the normalized particle counts (y-axis) of differentially phosphorylated tau proteins at the indicated molecular masses (x-axis). For each protein, the molecular mass was measured at three different indicated concentrations (indicated in z-axis). Dark lines indicate Gaussian fits to the peaks. The total particle counts for each protein at corresponding concentrations: *E. coli* GFP-tau at 1 nM: *N*  =  197, at 5 nM: *N*  =  460, at 25 nM: *N*  =  943. HEK GFP-tau at 1 nM: *N*  =  144, at 5 nM: *N*  =  1092, at 25 nM: *N*  =  2657. Sf9 GFP-tau at 1 nM: *N*  =  145, at 5 nM: *N*  =  770, at 25 nM: *N*  =  2361. Molecule landing events were collected over two independent experiments for each condition: *E. coli* GFP-tau at corresponding concentrations: *n* =  3. HEK GFP-tau and Sf9 GFP-tau at corresponding concentrations: *n* =  2. The calculated mass, percentage of particles, and total particle counts of each oligomeric state are indicated. Cartoons indicate the hypothesized oligomeric state of each indicated peak. The theoretical molecular weight of GFP-tau is indicated.

Hyperphosphorylation of tau has been implicated in its oligomerization and the pathogenesis of neurodegenerative tauopathies^20–24^. Previous studies have demonstrated that tau hyperphosphorylation in Sf9 and HEK cells enhances oligomerization^71,72^. To more precisely characterize the oligomeric state of differentially phosphorylated tau proteins, we employed mass photometry across a range of nanomolar tau concentrations. For these measurements, tau proteins retained their N-terminal sfGFP tag, which shifts the expected monomer mass well into the instrument's robust detection range. sfGFP has been extensively characterized as a robustly monomeric, soluble fusion partner that does not drive non-specific oligomerization of its fusion targets at the concentrations used here^73^, and the observation that the three tau variants produced distinct oligomerization profiles despite carrying the identical sfGFP tag confirms that the species we detect reflect tau-tau interactions rather than tag-driven multimerization. Recombinantly expressed tau from *E. coli* remained predominantly monomeric ( ~ 95%) across a 25-fold concentration range, with no detectable dimer formation (Fig. 4). Both HEK- and Sf9- derived tau existed primarily as monomers at 1 nM, with no observable dimers. However, increasing the concentration to 5 nM promoted oligomerization, resulting in 22% and 11% dimer formation for HEK and Sf9 tau, respectively (Fig. 4). At 25 nM, dimeric species increased to 32% and 12% for HEK and Sf9 tau, respectively, with HEK tau also exhibiting 4% trimer formation (Fig. 4). These findings corroborate previous biochemical studies demonstrating increased oligomerization propensity of Sf9-expressed phosphorylated tau^71^ and highlight the increased tendency of HEK-derived tau to form higher-order soluble oligomers at elevated concentrations. Differential phosphorylation patterns likely contribute to the observed variations in oligomerization behavior between HEK and Sf9 tau and require further investigation. These results demonstrate that tau phosphorylation favors the formation of small soluble tau oligomers starting with dimers and trimers.

### Pathological tau phosphorylation pattern facilitates the assembly of full-length tau into CTE-like NFT fibrils

**Fig. 5:**
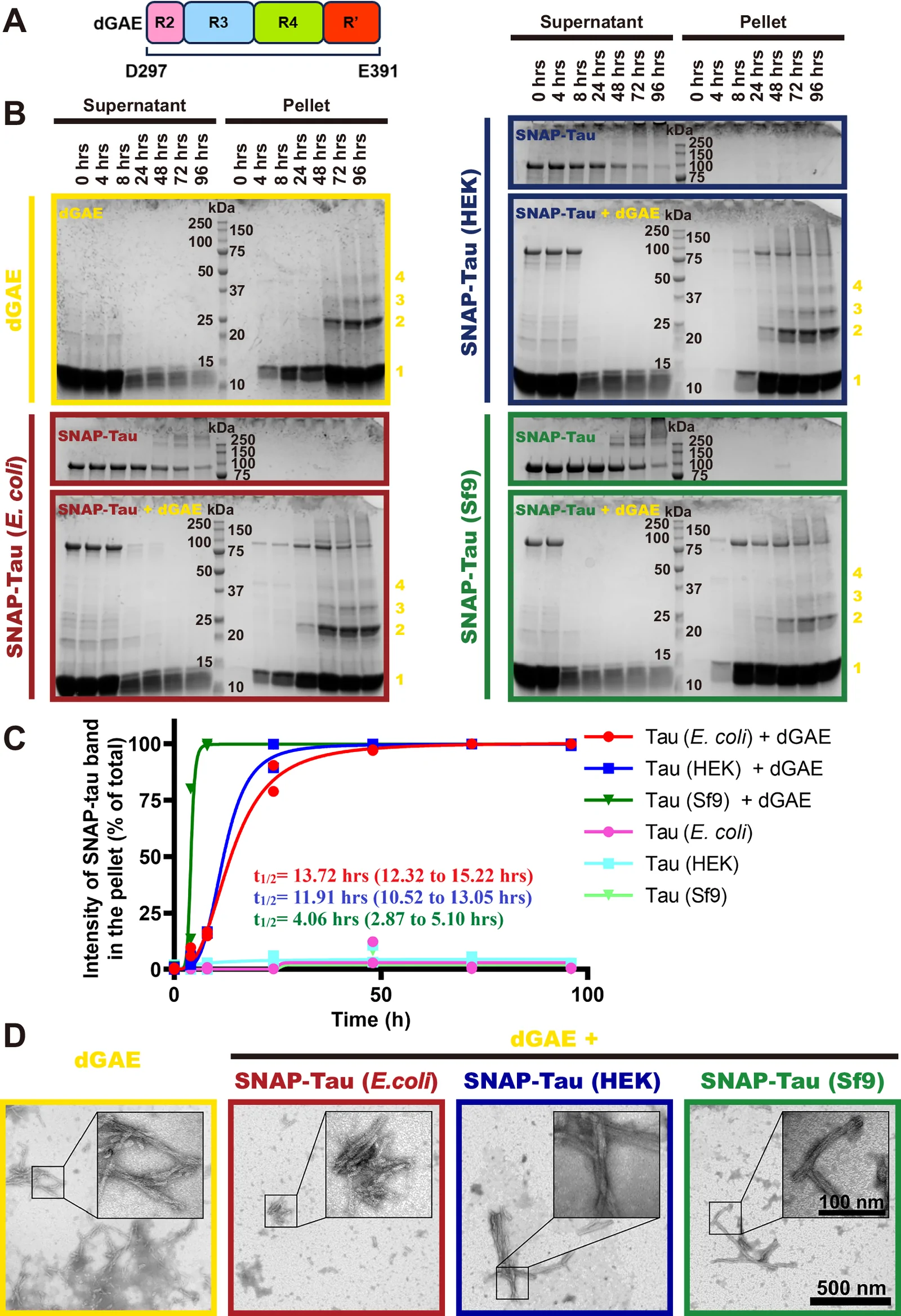
Specific phosphorylation patterns promote tau co-assembly into CTE-like fibrils formed by the tau fragment dGAE. **A** Diagram of dGAE, which contains a C-terminal portion of R2, intact R3, R4 and the N-terminal portion of R’. **B** Coomassie-stained SDS-PAGE gels showing the assembly kinetics of CTE-like fibrils indicated by pelleting via centrifugation. dGAE bands representing inferred oligomeric states are indicated by numbers indicating those states (1: monomer, 2: dimer, 3: trimer, etc.). Molecular weight markers for all gels are indicated. For quantification, the protein intensities of full-length tau proteins, including high-order oligomers, in both supernatants and pellets at corresponding timepoints were quantified. For each SNAP-tau, two independent experiments were performed. **C** Quantification of the co-assembly kinetics of the differentially phosphorylated tau proteins. The fractions of the differentially phosphorylated full-length tau proteins in the pellet at the indicated time points in each condition were evaluated by comparing intensities of full-length tau in the pellet versus in total (pellet + supernatant) in each condition. Two independent experiments (*n* = 2) were performed. Curves were fitted to one-site-specific binding with Hill slope. The calculated *t*_*1/2*_ and the corresponding 95% confidence intervals (95% CI, in parentheses) are indicated. **D** Negative-stain transmission electron microscopy (TEM) images of filaments assembled from dGAE or co-assembled from dGAE + differentially phosphorylated full-length tau proteins. Scale bar: 500 nm. Square enclosed regions showing high magnification views of the assembled filaments. Scale bar: 100 nm.

A common feature of tauopathies is the intracellular accumulation of NFTs composed of hyperphosphorylated tau^20–25^. While hyperphosphorylated tau is a major component of these filaments, the causal relationship between tau phosphorylation and NFT assembly remains a subject of ongoing debate^39^. Recent studies have achieved the in vitro assembly of tauopathy-relevant filaments without the need for polymerization-promoting cofactors^50,51,74,75^. To investigate the role of tau phosphorylation in filament assembly, we conducted an in vitro polymerization assay to generate tau fibrils that adopt the same conformation as those isolated from brains with tauopathy. In this assay, we used a tau fragment derived from the AD/CTE tau filament core, spanning residues 297–391 of the protein^26,28^. This fragment, known as dGAE^76^, encompasses intact regions of the microtubule binding repeat R3 and R4, as well as part of R2 and the pseudo-repeat (Fig. 5A). Previous studies have demonstrated the successful polymerization of dGAE into AD-like and CTE-like filaments in vitro, in the absence of aggregation-inducing cofactors, which exhibit identical atomic structures to those isolated from AD and CTE brains^50,51^.

We formed CTE-like filaments by continuous agitation of 400 μM dGAE in 10 mM PB (pH 7.4), 10 mM DTT, and 200 mM NaCl^50,51^. Analysis by ultracentrifugation and SDS-PAGE revealed assembly initiation before 4 h, detectable higher-order oligomers at 24 hours, and near-complete incorporation of dGAE into filaments by 96 h (Fig. 5B). Negative-stain transmission electron microscopy (TEM) confirmed the characteristic twisted filament structure consistent with previous reports of CTE-like filaments (Fig. 5D)^50,51^.

To assess the impact of phosphorylation on the incorporation of tau molecules into the growing filaments, differentially phosphorylated full-length SNAP-tagged tau proteins were co-assembled with dGAE. A 1:99 molar ratio of SNAP-tau (4 μM) to dGAE (396 μM) was used to minimize any steric effects from the SNAP tag^77^. Parallel assays with SNAP-tau alone (4 μM) served as controls. Ultracentrifugation and SDS-PAGE analysis revealed near-complete incorporation of all differentially phosphorylated SNAP-tau species into the pellet fraction after 96 h of co-assembly, whereas in the absence of dGAE, full-length tau remained in the supernatant (Fig. 5B, C). Because SNAP-tau alone did not pellet under these conditions, and because incorporation kinetics differed strongly among the three tau preparations, the recovered pellet fraction is unlikely to reflect only nonspecific trapping by dGAE aggregates. Negative-stain TEM confirmed the formation of twisted filaments in all co-assembly samples after 96 h (Fig. 5D), indicating incorporation of the differentially phosphorylated tau into CTE-like filaments in the presence of dGAE, while full-length tau alone did not assemble under these conditions.

To quantify the impact of phosphorylation on aggregation kinetics, the fraction of pelleted full-length tau was determined at each time point and fitted using the Hill equation (Fig. 5C). The t_1/2_ values for *E. coli* tau and HEK tau were similar ( ~ 14 h and ~12 h, respectively). However, the t_1/2_ for Sf9 tau was significantly faster ( ~ 4 h, Fig. 5C), demonstrating that the Sf9 tau phosphorylation pattern strongly promotes co-assembly into CTE-like filaments. While both HEK and Sf9 tau exhibit similar overall phosphorylation levels ( ~ 5-6 phosphates per molecule), their phosphorylation patterns differ. Given the reported similarity between the phosphorylation pattern of insect cell-expressed tau and AD-derived tau^49^, Sf9 tau appears to be a robust pathological tau model system. Our co-assembly assay reveals that particular phosphorylation patterns facilitate the assembly of full-length tau molecules into pathologically relevant fibril structures.

## Discussion

Tau phosphorylation is a critical regulator of both physiological and pathological processes in mammals. Physiologically, tau phosphorylation is implicated in diverse functions, including development^44–46^, hibernation^41–43^, normal sleep^78^, and anesthesia-induced modulation of consciousness^79,80^. During these processes, transient increases in tau phosphorylation at multiple sites are reported, which subsequently return to lower physiological levels^41,43,47,78^. Conversely, pathologically altered tau phosphorylation disrupts microtubule (MT) binding affinity and induces conformational changes, potentially leading to tau mislocalization from axons to the cytosol^40^ or promoting aggregation^81^. Consistent with this, highly phosphorylated tau is a major component of paired helical filaments (PHFs) in several tauopathies^20,24,25^. Intriguingly, while physiological tau phosphorylation appears reversible, that observed in pathological conditions appears to be irreversible, despite similarities in phosphorylation levels between fetal and Alzheimer’s disease (AD) tau^39,45,46,48^. Furthermore, the functional consequences of phosphorylated tau in physiological contexts and the precise timing of tau phosphorylation relative to insoluble aggregate formation in AD and other tauopathies remain unclear^39^.

To elucidate the role of tau phosphorylation, we used two distinct forms of heterogeneously phosphorylated tau, which we argue more closely mimic the complexity of in vivo states than tau with single, specific phospho-modifications. Our models included HEK tau, which is derived from human embryonic kidney (HEK) 293 cells and represents a physiological-like phosphorylation pattern and Sf9 tau, which exhibits a phosphorylation pattern similar to that found in AD brains^49^, and thus serves as a model for pathological tau. This approach allows us to probe the functional consequences of a pathologically relevant ensemble of phosphorylation, which is critical given that tau in AD brains is also known to be heterogeneously phosphorylated^64^. While we recognize that other post-translational modifications are potentially present on our cell-derived tau, our study was designed to specifically isolate and characterize the functional consequences of the distinct phosphorylation patterns, which are central to tau’s regulation and pathology. Our LPP rescue experiments show that phosphorylation is the dominant driver of the phenotypes observed for Sf9 tau, although they do not exclude a minor residual contribution from other PTMs.

A related consideration is how the phosphorylation state of our Sf9 tau model compares to that observed in the AD brain. Near-complete phosphorylation at certain sites, such as those within the S196-S210 cluster, is primarily a feature of insoluble, late-stage NFT-incorporated tau isolated from end-stage AD tissue, where high-occupancy modification across the proline-rich region and C-terminus has been well documented^64^. By contrast, the soluble hyperphosphorylated tau pool that precedes filament assembly, and which is arguably the most disease-relevant intermediate for understanding pathological conversion, exhibits a more heterogeneous and substoichiometric phosphorylation profile that more closely resembles our Sf9 tau model. Our Sf9 tau, carrying approximately 6 phosphorylated sites per molecule, falls within the 5–9 site range reported for pathological tau in tauopathy brains, as compared to the 2-3 sites typical of healthy adult brain tau^66^. This interpretation is further supported by recent evidence that phosphorylation at MARK kinase target sites S262 and S356 marks prefibrillar, granular tau aggregates in pre-NFT neurons and is largely absent from mature ThS-positive tangles, suggesting these sites represent early initiating rather than terminal phosphorylation events^82,83^. Our model, therefore, captures the phosphorylation landscape most relevant to the early stages of pathological tau conversion.

Previous studies have suggested that the addition of phosphate groups can induce local charge effects, which in turn alter the conformation and molecular interactions of tau^84,85^. In our study, we observed that HEK tau and Sf9 tau exhibit similar phosphorylation levels, with approximately 5-6 phosphorylated sites per molecule. As a result, both tau preparations showed reduced affinity for MTs compared to non-phosphorylated tau (Fig. 2), consistent with previous findings that specific phosphorylation at sites S214, T231, S235, or S262 inhibits MT interactions (Fig. 1C)^32,34^. However, despite this similarity, Sf9 tau displayed a significantly lower microtubule affinity than HEK tau, which might be attributed to the higher phosphorylation levels at S237 and S352 in Sf9 tau (Fig. 1C). Specifically, the phosphorylation at S237 is at least 2-fold higher than in HEK tau, which is noteworthy given that phosphorylation of S235, a nearby residue, has been shown to inhibit microtubule binding^32^. Additionally, S352 is located in a conserved 12-residue stretch within R4 of the microtubule binding repeats, the corresponding residue in R1 (S258), has been shown to form a hydrogen bond with α-tubulin E434^70^, perhaps hinting at a mechanism for phosphorylation-induced disruption of microtubule binding. The corresponding residue S352 in R4 may also form a hydrogen bond with α-tubulin E434; the phosphorylation of S352 may also disrupt the interaction between tau and tubulin. Furthermore, S352 is located within the microtubule binding repeat R4 (Fig. 1C), which is proximal to Ser356 within the KXGS motif. The KXGS motifs are found in the microtubule binding repeats of tau, MAP2 and MAP4 (X = I or C). These KXGS motifs are targets of the microtubule-affinity regulating kinase (MARK)^30,86–88^. Previous experiments have demonstrated that phosphorylation within these motifs reduces MAP affinity for microtubules^30,87–89^. These findings suggest that the distinct phosphorylation patterns, and therefore charge distributions, between HEK tau and Sf9 tau may contribute to their differing microtubule affinities.

Despite reduced MT affinity, both HEK and Sf9 tau retained the ability to form tau envelopes and compact the MT lattice in vitro (Fig. 3C, E), suggesting the preservation of this crucial tau function even in pathologically phosphorylated tau. This observation is consistent with previous data showing that both *E. coli* and Sf9 tau form envelopes along microtubules, albeit at different concentrations^13,56^. To compensate for its reduced affinity, we found that Sf9 tau exhibited increased cooperative interactions on MTs (Fig. 3B). The molecular mechanism underlying this enhancement remains unclear. We note that S352, located within R4 of the MTBR and phosphorylated at ~45% occupancy in Sf9 tau but not in HEK tau (Supplementary Table 1), represents one candidate site whose modification could alter the local charge environment of the repeat domain in ways that promote tau–tau contacts at the microtubule surface. Additionally, the high-occupancy Sf9-specific C-terminal modifications at S396, S404, and S422 (Supplementary Table 1) may contribute by influencing tau’s overall conformational ensemble and its propensity to engage neighboring tau molecules. These findings suggest that phosphorylation does not abolish microtubule-associated tau activities, even though simple microtubule binding alone is not a complete measure of tau physiological function. Instead, our data support the view that phosphorylated tau can still participate in microtubule-based regulation through envelope formation and lattice remodeling, while future work will be needed to test other outputs, such as tubulin assembly, in more native contexts.

The observation that HEK tau shows impaired cooperative dwell times within non-phosphorylated *E. coli* tau envelopes (Fig. 2E), yet retains a Hill coefficient essentially identical to that of *E. coli* tau when forming its own envelopes (Fig. 3B), is intriguing. These results suggest that the functional consequences of HEK tau phosphorylation on cooperative tau-tau interactions are sensitive to the phosphorylation context of neighboring molecules on the microtubule. The molecular basis of this context-dependence remains unclear, and future work, including high-resolution structural studies of phosphorylated tau envelopes, will be needed to resolve it.

We observed that both HEK and Sf9 tau displayed concentration-dependent oligomerization in solution at nanomolar concentrations (Fig. 4), indicating that phosphorylation of tau favors the formation of small soluble tau oligomers in solution. Given the relatively high reported abundance of tau in the neuronal cytoplasm ( ~ 2 μM)^39^ and the presence of 2-3 phosphorylation sites in healthy brains^66,67^, our findings suggest that small tau oligomers may be present in the cytoplasm under physiological conditions. Consistent with this, a recent study shows that tau can form dimeric and trimeric complexes under physiological conditions in cultured neurons and under non-aggregated conditions in BSC-1 and QBI-293 cell lines^90^.

In our fibril co-assembly assays, Sf9 tau more readily co-assembled with the dGAE to form CTE-like filaments than either HEK or *E. coli* tau (Fig. 5B, C). Given the similar overall phosphorylation levels between Sf9 and HEK tau, our results suggest that the pattern of phosphorylation, rather than overall level, is a crucial determinant for tauopathy-relevant filament assembly. Specifically, enhanced phosphorylation at S210 and S422 in Sf9 tau may facilitate co-assembly, as these sites are more highly phosphorylated in Sf9 tau than HEK tau, and phosphorylation at both sites is associated with AD (Fig. 1C)^61–65^. Furthermore, phosphorylation at S208, near S210, as well as S422, is known to promote aggregation^35,36,38^. Phosphorylation at S262, which inhibits in vitro aggregation of a truncated tau fragment that encompasses the microtubule-binding repeats (termed K18)^91^, was also detected in our HEK tau preparation (Fig. 1C), likely contributing to its reduced co-assembly propensity. While our current work focuses on the emergent properties of phosphorylation patterns, we suggest that further site-specific mutagenesis will be crucial to define the precise roles of these individual sites. Such studies represent an important future direction that will build upon the foundational framework established here. Importantly, neither of the differentially phosphorylated tau proteins formed filaments independently under the low-concentration conditions we tested. However, we find that phosphorylation promotes oligomerization of tau in solution at nanomolar concentrations (Fig. 4), underscoring the potential for phosphorylation to play multiple roles in tau aggregation, both promoting oligomerization and influencing filament formation in possibly distinct ways. Deciphering the precise mechanisms of these processes will be the focus of future research.

Our mass photometry data demonstrate that phosphorylated tau proteins exhibit a heightened propensity to form small oligomers in solution. Furthermore, co-assembly assays revealed that phosphorylated Sf9 tau more readily incorporates into large fibrils when combined with dGAE. These findings raise an intriguing question: is there a mechanistic link between tau oligomerization and fibrillization, or do these soluble oligomers represent an initial step in the fibrillization pathway? Interestingly, our assays indicate that phosphorylated HEK tau, despite exhibiting the highest oligomerization propensity in solution, did not show significantly different co-assembly properties from non-phosphorylated *E. coli* tau. This suggests that the small oligomers formed by phosphorylated tau may not necessarily serve as the nucleation point for tau fibrillization, consistent with a previous study^90^, which found that physiological tau oligomers (dimers and trimers) are distinct from pathological aggregates with respect to phosphorylation state. Our work further highlights this distinction by revealing that specific phosphorylation patterns dictate the susceptibility of tau to transition into the aggregated state. Given that HEK tau and Sf9 tau in our study likely adopt different conformations in solution (Figs. 1 and 4), we propose that molecular conformation, rather than the overall phosphorylation status, is a critical determinant in tau filament assembly. This highlights the importance of conformational dynamics in regulating tau aggregation and fibrillization pathways.

**Fig. 6:**
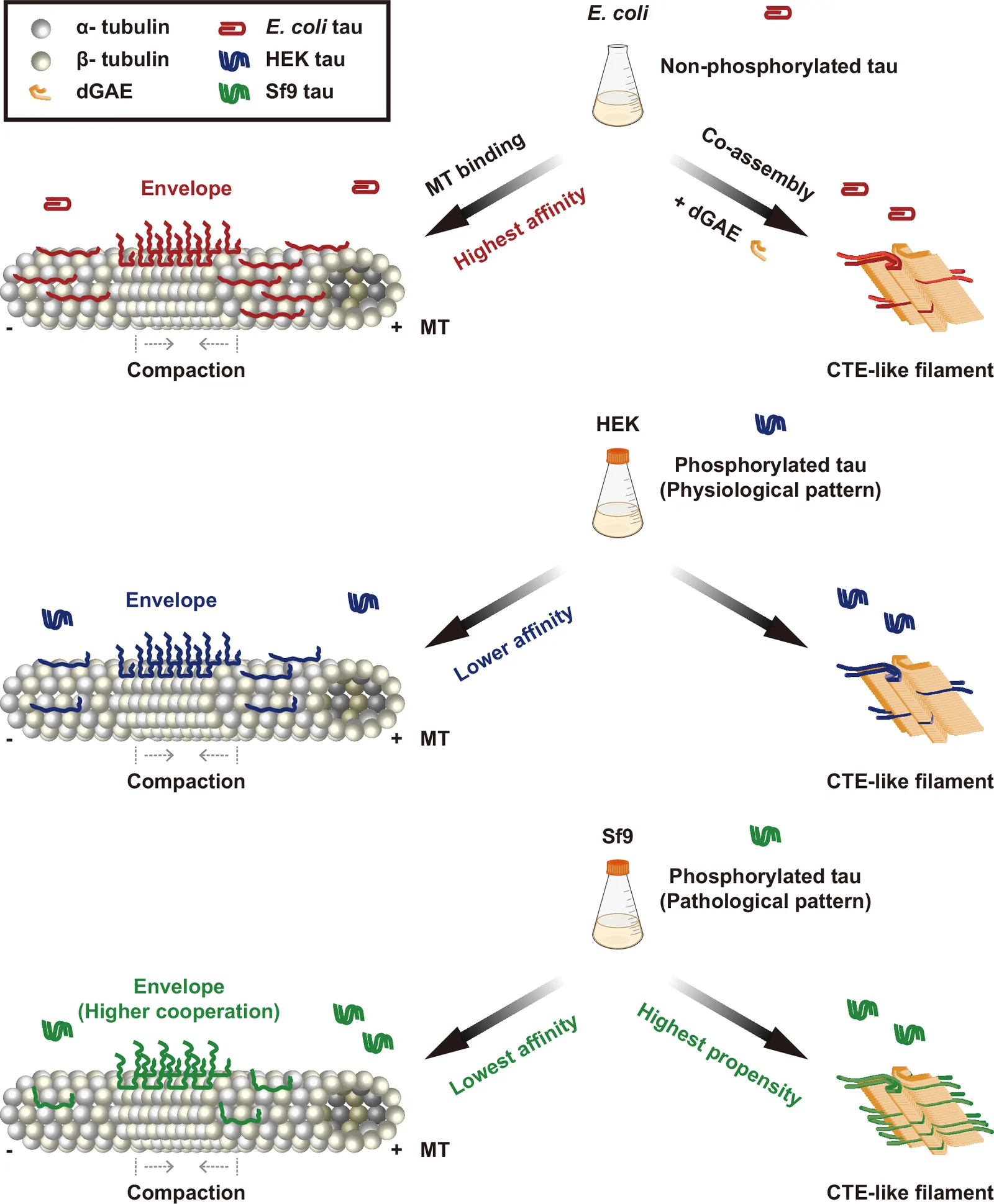
Summary of effects of tau phosphorylation on microtubule interactions and the tauopathy fibril co-assembly. Schematic showing a model for the effect of tau phosphorylation on microtubule interactions and CTE-like filament co-assembly. Middle panels showing the recombinant human 2N4R tau proteins were expressed and purified from different cellular sources: *E.coli* tau represents a non-phosphorylated protein, HEK tau represents a physiological-like phosphorylation pattern, and Sf9 tau represents a pathological-like phosphorylation pattern. Left panels showing the interactions between the microtubule and differentially phosphorylated tau proteins. Although phosphorylation impairs the overall microtubule affinities of tau, all three types of tau proteins can form cooperative envelopes along microtubules and retain the ability to compact the microtubule lattice. Sf9 tau displayed the lowest microtubule affinity, but conversely, the highest cooperative interactions during the envelope formation. Adjacent panels showing the role of tau phosphorylation in CTE-like filament co-assembly. Sf9 tau displayed the highest propensity for CTE-like filament co-assembly, indicating that the phosphorylation pattern, but not the total level of phosphorylation, dramatically alters the propensity of full-length tau molecules to enter the pathological conformation.

In summary, our study revealed that the pathological phosphorylation pattern facilitates the cooperative interaction of tau molecules along microtubules, potentially as a way to compensate for reduced microtubule affinity. Additionally, the pathological phosphorylation pattern also promotes tau molecules to co-assemble with dGAE into CTE-like filaments (Fig. 6). Our study provides insights into the role of tau phosphorylation in regulating tau microtubule interactions and envelope formation, oligomerization, and tauopathy-related filament assembly. We find that the pattern of phosphorylation, rather than the overall level, is a key determinant of tau susceptibility to transition from a highly soluble, unstructured molecule to pathologically relevant filament assembly states.

## Methods

### Plasmids

A Full-length human tau cDNA-containing plasmid was purchased from Addgene (16316). Coding sequences of Full-length human 2N4R tau or its truncated fragment dGAE (aa297-391) were amplified by PCR and subsequently cloned into a pET28a vector (Invitrogen) or a pFastBac vector (Invitrogen) or a pAceMam2 vector (GenevaBiotech) containing either an N-terminal His_6_- StrepII-sfGFP or His_6_-StrepII-SNAPf by Gibson^13,92^. To increase the gene expression, the pAceMam2 vector was further optimized by adding a woodchuck hepatitis virus post-transcriptional regulatory element (WPRE) downstream of the ORF^93^. All constructs were verified by Sanger sequencing.

### Protein expression and purification

dGAE, StrepII-sfGFP-tau and StrepII-SNAPf-tau in pET28 were expressed in BL21-CodonPlus (DE3)-RIPL cells (Agilent). Briefly, a single colony was inoculated into 50 mL LB Broth medium (Fisher Scientific) and grew overnight at 37 °C, the culture was then diluted 1 to 500 into 500 mL LB Broth medium (Fisher Scientific) and cultured at 37 °C until the optical density at 600 nm (OD600) reached 0.5. Expressions were induced 18 h by 0.4 mM isopropyl-β-d-thiogalactoside (Fisher Scientific) at 24 °C for dGAE and StrepII-sfGFP-tau, for StrepII-SNAPf-tau, the expression was induced 4 h by 0.4 mM isopropyl-β-d-thiogalactoside (Fisher Scientific) at 37 °C. Cells were subsequently harvested by centrifuge at 1000×g for 10 min and flash frozen by liquid nitrogen. All frozen cells were stored at −80 °C before purification.

StrepII-sfGFP-tau and StrepII-SNAPf-tau in pFastBac were expressed in insect cells. Briefly, insect Sf9 cells were grown or maintained in Sf-900 II serum-free medium (Thermo Fisher Scientific) at 27 °C. Plasmid DNA of corresponding constructs was transformed into DH10EmBacY competent cells (Geneva Biotech) to generate bacmids. To get baculovirus, 1 × 10^6^ Sf9 cells were transferred to each well of a six-wells plate and subsequently transfected with 1 μg of bacmid DNA by 6 µL of Cellfectin (Thermo Fisher Scientific). Baculovirus-containing cell supernatants (P1) were harvested 7 days after transfection when the entire cell population has become infected. To amplify the baculovirus (P2), 50 mL of Sf9 cells were infected with 50 µL of P1 virus, and the P2 virus was harvested 5 days after infection. To prepare recombinant proteins, 200 mL of Sf9 cells at high density ( ~ 2 × 10^6^ cells/ml) were infected by P2 virus (virus: cells  =  1:100, v/v) and cultured around 65 h at 27 °C. Cells were subsequently harvested by centrifuge at 1000 × g for 10 min and flash frozen by liquid nitrogen.

StrepII-sfGFP-tau and StrepII-SNAPf-tau in pAceMam2 were expressed in mammalian cells. Briefly, suspension-adapted human embryonic kidney (HEK) cell line FreeStyle 293-F cells (Invitrogen) were grown or maintained in FreeStyle™ 293 Expression Medium (Thermo Fisher Scientific) at 37 °C with a humidified atmosphere of 8% CO_2_. To prepare recombinant proteins, 400 mL of 293-F cells at high density ( ~ 1 × 10^6^ cells/ml) were transiently transfected by corresponding plasmid DNA (v_cells_: m_DNA_ = 1:1 mL/μg), the transient transfections were mediated by polyethyleneimine (PEI, m_DNA_: m_PEI_ = 1:4). The transfected cells were cultured around 72 h at 37 °C with a humidified atmosphere of 8% CO_2_. Cells were subsequently harvested by centrifuge at 1000 × g for 10 min and flash frozen by liquid nitrogen.

To purify the dGAE protein, the frozen pellet 1 L culture was thawed on ice and resuspended in 40 mL of wash buffer (WB: 50 mM MES pH 6.0, 10 mM EDTA, 10 mM DTT), freshly supplemented with 1 mM dithiothreitol (DTT), 1 mM phenylmethylsulfonyl fluoride (PMSF), protease inhibitor mix (Promega) and 5 µL of Benzonase Nuclease (Millipore). Cells were lysed by passing through an Emulsiflex C-3 (Avestin) and were centrifuged at 15, 000×g for 20 min at 4 °C to obtain soluble lysate. Clarified lysate was filtered through 0.45 μm filters and loaded onto a HiTrap SP HP 5 mL column (GE Healthcare) for cation exchange. Bound proteins were eluted with 30 mL of a linear gradient of 0 – 1 M NaCl in WB buffer. Protein-containing fractions were pooled and precipitated using 30% (m/v) ammonium sulfate and left on a rocker for 30 min at 4 °C. Precipitated proteins were then centrifuged at 20,000  ×g for 35 min at 4°C and resuspended in 2 mL of 10 mM PB (pH 7.4), freshly supplemented with 10 mM DTT, and loaded onto a 60/10 Superdex 200 size exclusion column. Size exclusion fractions were analyzed by sodium dodecyl sulfate-polyacrylamide gel electrophoresis (SDS-PAGE), protein-containing fractions pooled and concentrated to 6 mg/ml using molecular weight concentrators with a cut-off filter of 3 kDa. The dGAE concentration was measured by NanoDrop One (Thermo Fisher Scientific) based on the UV absorbance at 280 nm using an Abs 0.1% of 0.144 and was further confirmed by comparing with the standard bovine serum albumin (BSA) concentrations on SDS-PAGE gel. Purified protein samples were flash frozen in 100 μL aliquots for future use.

To purify StrepII-sfGFP-tau and StrepII-SNAPf-tau, frozen pellets were thawed on ice and resuspended in 40 mL of purification buffer (50 mM Tris–HCl pH 8.0, 150 mMKCH_3_COO, 2 mM MgSO_4_, 1 mM EGTA, 5% glycerol) freshly supplemented with 1 mM dithiothreitol (DTT), 1 mM phenylmethylsulfonyl fluoride (PMSF), and protease inhibitor mix (Promega). Resuspended cells were lysed by passing through an Emulsiflex C-3 (Avestin) (for *E. coli* expression) or were homogenized in a dounce homogenizer and lysed by the addition of 1% Triton X-100 and 5 µL of Benzonase Nuclease (Millipore) for 10 min on ice (for insect cell and mammalian cell expression). To obtain soluble lysate, lysed cells were centrifuged at 15,000 × g for 20 min at 4 °C. The clarified lysate was incubated with 10 mL of Strep-Tactin XT resin (IBA) for 1 h at 4 °C and washed with purification buffer, protein was subsequently eluted by elution buffer (purification buffer plus 100 mM biotin, pH 8.0). Eluted proteins were subsequently diluted 20x by IEX Buffer pH 8.0 (Ionic Exchange Buffer: 50 mM Tris-HCl pH 8.0, 2 mM MgCl_2_, 1 mM EGTA and 10% glycerol) and were loaded onto a 5 mLHiTrap Q HP column (Cytiva), bound proteins were eluted with a linear gradient of 0–1 M NaCl in IEX Buffer. Eluted protein was further purified by Superdex 200 columns (Cytiva) in PB buffer. Protein-containing fractions were pooled and supplemented with 10% Glycerol. Protein concentrations were measured by NanoDrop One (Thermo Fisher Scientific) based on the absorbance of the attached fluorophores (StrepII-sfGFP-tau) or the UV absorbance at 280 nm using an Abs 0.1% of 0.550. All protein aliquots were subsequently flash frozen by liquid nitrogen and stored at −80 °C.

To purify tau for cleavage, *E. coli* and SF9-derived tau was expressed as mentioned above, with the exception that SF9 tau was expressed in 600 mL culture at 1 × 10^6^ cells/mL. Cells were resuspended with His Buffer (50 mM Tris-HCl pH 7.5, 300 mM NaCl, 15 mM Imidazole) freshly supplemented with 2 mM PMSF and 2x protease inhibitor mix (Promega). Resuspended cells were then lysed and clarified as described above. Clarified lysate was then bound on a 10 mL Ni-NTA resin (Invitrogen) column by gravity flow. Once lysate had bound, columns were washed with 5x CV of His Buffer. Once the column has been thoroughly washed, the Ni-NTA resin was incubated with 0.26 mg Prescission Protease (Cytiva) for 16 hr with gentle agitation on a tube roller at 4 °C. Cleaved proteins were then collected and diluted ~6-10x on with CIEX Buffer A(Cleaved IEX Buffer: 50 mM NaPO_4_ pH7). Diluted proteins were filtered and loaded onto a 1 or 5 mL HiTrap SP HP column (Cytiva). Proteins were eluted with a linear gradient of 0 - 1 M NaCl in CIEX buffer. Proteins containing fractions were pooled and concentrated to run on the Superdex 200 column in GF150 buffer (25 mM HEPES-KOH pH 7.4, 150 mM KCl, 2 mM MgCl_2_).

Full uncropped Coomassie gels for all purified tau preparations are provided in Supplementary Figs. 3 through 10.

### Cell line authentication

The FreeStyle 293-F cells (cat. no. R79007) and the Sf9 cells (cat. no. 11496015) used in this study were obtained from Thermo Fisher Scientific. Cell lines were not independently authenticated beyond the vendor's certification at the time of purchase. Cell lines were not tested for mycoplasma contamination in our laboratory. Neither FreeStyle 293-F nor Sf9 cells appear in the ICLAC register of commonly misidentified cell lines.

### Labeling differentially phosphorylated tau proteins with SNAP tag substrate

Differentially phosphorylated StrepII-SNAP-tau proteins were fluorescently labeled with SNAP-Surface 649 (NEB) according to the manufacturer’s protocols. Briefly, differentially phosphorylated StrepII-SNAP-tau proteins were diluted to ~ 25 μM by purification buffer (50 mM Tris–HCl pH 8.0, 150 mM KCH_3_COO, 2 mM MgSO_4_, 1 mM EGTA, 5% glycerol), then incubated with 50 μM of SNAP-Surface 649 on ice for 2 h. Excess SNAP-Surface 649 was removed by 5 mL HiTrap Desalting column (Cytiva) equilibrated with PB buffer.

### Dephosphorylating SF9 expressed Tau

Purified SF9 expressed sfGFP-Tau was diluted to 2 μM in a 25 μL solution containing 1x Protein Metallophosphatase buffer (NEB), 1 mM MnCl_2_ (NEB), and 400 units of Lambda Phosphatase (NEB). The reaction mixture was then incubated at 30 °C for 1 h. Dephosphorylated proteins were then either used immediately or flash frozen and stored in −80 °C until further use.

### Mass spectrometry

Protein samples were treated with standard tryptic digestion. Briefly, samples were reduced in 5.5 mM DTT at 56 °C for 45 min, followed by alkylation with iodoacetamide added to a final concentration of 10 mM for one hour. Subsequently, samples were digested overnight by trypsin at a final mass ratio of 1:50 (enzyme: substrate) at 37 °C. The reaction was quenched by flash freezing in liquid nitrogen, and the digest was lyophilized. Digests were reconstituted in 0.1% TFA with 10% acetonitrile prior to injection.

Two batches of tryptic digests were analyzed using different instruments. The first batch of tryptic digests was analyzed using a nanoAcquity UPLC system (Waters) connected to the Xevo G2 QTof mass spectrometer. Samples were loaded onto a C18 Waters Trizaic nanotile of 85 μm × 100 mm; 1.7 μm (Waters). Mass spectrometry data were recorded for 60 min for each run and controlled by MassLynx 4.2 SCN990 (Waters). The acquisition mode was set to positive polarity under resolution mode. The second batch of tryptic digests was analyzed by liquid chromatography–tandem mass spectrometry on an Orbitrap Exploris 480 mass spectrometer (Thermo Fisher Scientific), Peptides were separated over a 30-minute analytical gradient prior to data-independent acquisition (DIA) using a Dionex Ultimate 3000 on a Pepsep 10 cm×150 um reverse phase column and a Thermos C18 trap column. Spectra were acquired in DIA mode with the Orbitrap serving as both MS1 and MS2 mass analyzer. Full MS1 survey scans were collected at a resolving power of 120,000 (empirical ~112,000–113,000). Precursors were fragmented across 34 sequential DIA isolation windows of 23 m/z width with 1 m/z overlap, spanning the precursor range of approximately 356.5–1105.5 m/z, and MS2 spectra were acquired at a resolving power of 15,000 (empirical ~14,100). The resulting duty cycle was approximately 1.56 s (MS1 and MS2). Maximum injection times were approximately 25 ms for MS1 and 22 ms for MS2. Spectra were recorded in profile mode and centroided on acquisition.

RAW MS files acquired from Waters instrument were processed using ProteinLynx Global Server (PLGS) version 3.0.3 (Waters, Milford, MA). Processing parameters consisted of a low energy threshold set at 200.0 counts, an elevated energy threshold set at 25.0 counts, and an intensity threshold set at 1500 counts. Each sample was searched against a customized human Tau protein database. Trypsin/LysC was selected as the enzyme. Carbamidomethylation was set as a fixed modification on cysteine, while phosphorylation on serine, threonine, and tyrosine residues, as well as methionine oxidation, were included as variable modifications. A reversed-sequence decoy database generated within PLGS was used to estimate peptide and protein false discovery rates. Protein identifications were accepted only if they met a confidence threshold greater than 95%. We did not perform a comprehensive quantitative comparison of non-phosphorylation PTMs across preparations, so a minor residual contribution from other PTMs cannot be excluded. Phosphorylation site localization was determined at the single-residue level using PLGS scoring of fragment ion spectra. For peptides containing multiple phosphorylation sites (Ser/Thr/Tyr), PLGS assigns the site of modification based on the presence of site-determining diagnostic fragment ions (b- and/or y-ions) that localize the phosphate group to a specific residue within the peptide sequence. Localization confidence is derived from the agreement between observed and theoretical fragmentation patterns, with particular emphasis on fragment ions that differentiate between alternative phosphorylation sites. Only phosphorylation sites supported by high-confidence fragment ion evidence and meeting PLGS scoring thresholds were retained for further analysis. Raw files acquired from the Thermo instrument were processed in a library-free (directDIA + ) workflow using Spectronaut v18.7 (build 18.7.240506.55695; Biognosys AG, Schlieren, Switzerland) with the default BGS Factory Settings. Spectra were searched against a human UniProt reference proteome (20,385 entries), a single Tau protein sequence, and a universal contaminant database (381 entries) appended to the search. Quantification was performed at the MS2 level using integrated peak areas. Cross-run normalization was applied (automatic strategy) and interference correction was enabled (minimum of 2 MS1 and 3 MS2 ions). Precursors were filtered by identification (Q-value), and protein quantities were derived using the MaxLFQ algorithm with the top 1–3 peptides per protein group and top 1–3 precursors per peptide. Phosphosite localization was performed within Spectronaut using a localization probability cutoff of 0.75. The mass spectrometry proteomics data have been deposited to the ProteomeXchange Consortium via the PRIDE^94^ partner repository with the dataset identifier PXD080008.

Site-specific phosphorylation degrees were estimated by directly comparing the total ion intensities of a phosphopeptide/peptide pair at certain sites as described^54,55^. Briefly, for each phosphorylation site, all peptides containing the specific site, whether phosphorylated or not, were analyzed. Each peptide was assigned a relative intensity representing its abundance in the sample. The phosphorylation degree for a given site was calculated by dividing the summed relative intensities of phosphorylated peptides by the total relative intensities of both phosphorylated and non-phosphorylated peptides containing that site. Two batch samples were prepared for Sf9 tau and HEK tau, respectively, and each sample was analyzed separately, resulting in two phosphorylation degree values for each site.

### Mass photometry

The mass photometry assays were carried out as described^95^. Briefly, to prepare chambers, microscope cover glass (#1.5 24 × 50 mm, Deckgläser) was cleaned by 1 h sonication in Milli-Q H_2_O, followed by another hour sonication in isopropanol, cover glasses were then washed by Milli-Q H_2_O and dried by flame. CultureWell silicone gaskets (Grace Bio-Labs) were cut and washed by Milli-Q H_2_O, CultureWell silicone gaskets were then dried by filtered air and placed onto the freshly cleaned cover glasses providing four independent sample chambers. Samples and standard proteins were diluted in SRP90 buffer (90 mM HEPES, 10 mM PIPES, 50 mM KCH_3_COO, 2 mM Mg(CH_3_COO)_2_, 1 mM EGTA, 10% glycerol, pH  = 7.4). The SRP90 buffer was freshly filtered by a 0.22 µm filter before measurement. For calibration, standard proteins BSA (Sigma), Alcohol dehydrogenase (Sigma) and beta-amylase (Sigma) were diluted to 5 – 50 nM. The non-specific binding events of single molecules on cover glasses were scattered and the masses of these molecules were measured by the OneMP instrument (Refeyn) at room temperature. Data were collected at an acquisition rate of 1 kHz for 100 s by AcquireMP (Refeyn) and subsequently analyzed by DiscoverMP (Refeyn). For each concentration of recombinant protein, measurements were performed three times and repeated using two different protein preparations. Results from one representative measurement are shown in the figure.

### Microtubule preparation

Porcine brain tubulin was isolated using the high-molarity PIPES procedure and then labeled with biotin NHS ester or Dylight-405 NHS ester as described (https://mitchison.hms.harvard.edu/files/mitchisonlab/files/labeling_tubulin_and_quantifying_labeling_stoichiometry.pdf). Porcine brains were obtained as postmortem tissue from a local commercial abattoir and processed within approximately 4 h of death. The animals were raised and slaughtered for commercial food production, independently of this study. No pigs were owned, bred, housed, transported, handled, subjected to experimental procedures, or euthanized by the authors or UC Davis personnel. Consequently, housing conditions and slaughter procedures were managed by the commercial facility and were not controlled or recorded by the research team. As this study used postmortem tissue only and involved no procedures on live vertebrate animals, UC Davis IACUC approval was not required. To polymerize microtubules, 50 µΜ of unlabeled tubulin, 10 µM of biotin-labeled tubulin, and 3.5 µM of Dylight-405-labeled tubulin were incubated with 2 mM of GTP for 20 min at 37 °C. Polymerized microtubules were stabilized by the addition of 20 µΜ taxol and incubated additional 20 min. Microtubules were pelleted at 20,000 × *g* by centrifugation over a 150 µL of 25% sucrose cushion and the pellet was resuspended in 50 μL BRB80 (80 mM Pipes (pH 6.8), 1 mM MgCl_2_ and 1 mM EGTA) containing 10 µΜ taxol.

### Total internal reflection fluorescence (TIRF) assays

TIRF chambers were assembled from acid-washed glass coverslips as described (http://labs.bio.unc.edu/Salmon/protocolscoverslippreps.html), pre-cleaned slide, and double-sided sticky tape. Chambers were first incubated with 0.5 mg/ml PLL-PEG-biotin (Surface Solutions) for 5–10 min, followed by 0.5 mg/ml streptavidin for 5 min. Microtubules were diluted by BRB80 containing 10 µΜ taxol. Diluted microtubules were flowed into streptavidin-adsorbed flow chambers and incubated for 5–10 min at room temperature for adhesion. To remove unbound microtubules, chambers were subsequently washed twice with SRP90 assay buffer (described above). Purified tau protein was diluted to indicated concentrations in the assay buffer with an oxygen scavenging system composed of PCA/PCD/Trolox^96^. Then, the solution was flown into the glass chamber. To quantify the dwell times, events longer than three pixels were selected and quantified, and two independent experiments were performed for each condition.

For the SiR-tubulin assay, chambers were preincubated 10 min at room temperature with 2 µM of SiR-tubulin in SRP90 assay buffer after washing. Then, differentially phosphorylated tau proteins were diluted to indicated concentrations with 2 µM SiR-tubulin containing SRP90 assay buffer and were flown into the glass chamber, respectively. Movies and images were acquired using a NIS-Elements software-controlled (AR, Version 5.20.2) Nikon Eclipse Ti2 Microscope (1.49 numerical aperture, ×100 objective) equipped with a TIRF illuminator and Andor iXon charge-coupled device electron multiplying camera. Data were analyzed manually using ImageJ (Fiji), and statistical tests were performed in GraphPad Prism 10.

### CTE-like filaments co-assembly assay

CTE-like filaments were assembled by dGAE alone (400 µM) or co-assembled by 99% dGAE (396 µM) plus 1% full-length tau (4 µM), the method was slightly modified from previous publication^50,51,97^. Briefly, 300 µL of purified dGAE (400 µM) or 300 µL of dGAE (396 µM) plus differentially phosphorylated full-length SNAP-tau proteins (4 µM) were incubated in assembly buffer, respectively. The assembly buffer contained 10 mM PB (phosphate buffer: pH7.4), freshly supplemented with 10 mM DTT, 200 mM NaCl, 1 mM phenylmethylsulfonyl fluoride (PMSF), protease inhibitor mix (Promega) and 0.01% NaN_3_. Then the mixed samples were agitated by an MS 3 digital shaker (IKA) at 37 °C (750 rpm) for 96 hours. 40 µL of samples were taken at corresponding timepoints and were subsequently flash frozen by liquid nitrogen and stored at −80 °C for further using.

### Analysis co-assembly efficiency by SDS-PAGE

40 µL of co-assembled samples frozen at different timepoints were thawed and ultracentrifuged 100,000 rpm at room temperature for 20 min by using an Optima MAX-XP ultracentrifuge with a TLA-100.1 rotor (Beckman Colter). Supernatants were transferred to 500 µL Eppendorf tubes, and pellets were resuspended with 40 µL of PB buffer (pH7.4). Then 12 µL of supernatants and pellets were loaded and run on SDS-PAGE, followed by staining with Coomassie blue. The protein intensities of full-length tau proteins in both supernatants and pellets at corresponding timepoints were subsequently measured and compared by Image J.

### Negative-stain TEM

Samples from pellets after 96 h agitation were centrifuged 15,000 rpm at room temperature for 15 min, the supernatants were discarded, and then the pellets were washed three times with PB buffer (pH7.4) and resuspended with 40 µL of PB buffer. 5 μL of samples from resuspended pellets were incubated on a discharged carbon-coated grid at room temperature for 1 min, excess solution was subsequently removed by filter paper, the grids were negatively stained five times with 5 μL of 0.75% Uranyl Formate, 30 s per time. The grids were left to air dry for 10 min and then were examined on a JEOL 1230 Transmission Electron Microscope.

### Statistics and reproducibility

All TIRF images and SDS-PAGE analyses were performed manually using ImageJ (Fiji) and all statistical tests were conducted in GraphPad Prism 11. At least two repeats were performed for each experiment to ensure the consistency and reliability of the data. For dwell time quantification, events exceeding three pixels were selected and quantified. For quantifying co-assembly kinetics, the intensities of protein bands were analyzed using ImageJ (Fiji). In Figs. 2C, 3D, E and S2D, differences between the two groups were compared using an unpaired two-tailed t-test; P values are shown in the corresponding figures. More detailed sample sizes and statistics are included in the legends of the corresponding figures. For microscopy-based assays, the number reported in figure legends corresponds to the total number of individual molecules or microtubules quantified within each condition, and this is reported separately from the number of independently repeated experiments.

### Reporting summary

Further information on research design is available in the Nature Portfolio Reporting Summary linked to this article.

## Supplementary information

Transparent Peer Review file

Supplementary Information

Description of Additional Supplementary Files

Supplementary Data 1

Reporting Summary

## Data Availability

Source data underlying all main figure graphs and charts are provided as Supplementary Data 1. Uncropped gels from the main figures are found in Supplementary Figs. 3-10. The mass spectrometry proteomics data are available via ProteomeXchange with identifier PXD080008.
