# Supplementary material for: A pathological phosphorylation pattern enhances tau cooperativity on microtubules and facilitates tau filament assembly
Source: Commun Biol. 2026 Aug 21;9:1408. doi: 10.1038/s42003-026-10797-6 (PMC13714281; doi:10.1038/s42003-026-10797-6)
Supplement: Supplementary file 1 [file 42003_2026_10797_MOESM1_ESM.pdf]

nature portfolio

Peer Review File

A pathological phosphorylation pattern enhances tau cooperativity on microtubules and facilitates tau filament assembly

Corresponding Author: Dr Richard McKenney

This file contains all reviewer reports in order by version, followed by all author rebuttals in order by version.

Version 0:

Reviewer comments:

Reviewer #1

(Remarks to the Author)
Fan et al. set out to analyze the properties of tau proteins with different phosphorylation patterns, focusing on microtubule binding, cooperative protein envelope formation, and NFT filament assembly. Tau proteins were expressed in bacteria, Sf9 cells, or HEK293 cells. Bacterial tau proteins are not phosphorylated, and Sf9-tau and HEK293-tau contain more than 50 phosphorylation sites. Phosphorylation at most of those sites was lower than 15%, and 11 or 12 sites had more than 15% of phosphorylation occupancy. They found that Sf9-tau and HEK293-tau show less binding rate to microtubules and to the envelopes than bacterial tau. Sf9-tau and HEK293-tau form oligomers and co-assembled dGAE-formed fibrils more efficiently than bacterial tau. The authors argue that these results reveal new insights into how tau phosphorylation impacts tau's physiological roles on microtubules and its pathoconversion into NFTs.
Their findings are potentially interesting. However, their conclusions are not fully supported by their results. The tau proteins they used are heterogeneous, and the roles of phosphorylation in their different behavior are not clear. Methods and experimental conditions are not sufficiently described. Results should be interpreted more carefully without bias. Many more experiments would be needed to support their conclusions.

Major points:
1. Tau proteins from cells are heterogeneous. As they showed, Sf9 tau proteins have about 5-6 phosphorylated sites per molecule and 10% of the average phosphorylation degree (it is not clear whether this means 10% of tau proteins are phosphorylated, or 10% of Ser, Thr, or Tyr in a tau protein are phosphorylated), thus, they are a mixture of tau proteins with different phosphorylation patterns. Authors argue that Sf9 tau has a pathological phosphorylation pattern since phosphorylated sites in Sf9 tau include several AD-related sites (see minor comment 1); however, to analyze the roles of tau phosphorylation at AD-related sites, it would be more straightforward to use tau proteins with specific phosphorylation pattern. Further attempts to purify phosphorylated tau, such as in vitro phosphorylation or immunoprecipitation or site-specific mutagenesis of specific phosphorylation sites, would be required to test the roles of phosphorylation.

2. Tau proteins from cells have various post-translational modifications. The difference in post-translational modifications other than phosphorylation may contribute to the different behaviors of Sf9-tau and HEK-tau. Mass Spectrometry would have detected post-translational modifications; however, authors seem to ignore PTM other than phosphorylation. They should include such data and compare all post-translational modifications between Sf9 and HEK293.

3. Authors should describe the experimental methods and conditions in more detail. 'Tau envelope' is not a commonly used assay and needs explanation. In Figure 2 and Figure 3, different concentrations are used in each experiment for each tau. Are they 'single molecule concentration'? What is it and how were they determined? Concentration would affect Hill co-efficient and Kd. Is it okay to compare them from these experiments and conclude they are ‘very similar’?

4. Figure 3. Within the envelope, a tau protein would interact with other tau proteins and microtubules. Which interaction does the measurement reflect? Authors state that ‘Furthermore, the dissociation constant (Kd) values of E. coli tau, HEK tau, and Sf9 tau were 6.19 nM,17.64 nM, and 59.71 nM respectively (Figure 3B), confirming that phosphorylation decreases the microtubule affinity of tau protein, consistent with our single-molecule results (Figure 2).’; however, I thought 3B calculate interaction between HEK tau and envelope tau. What is 'cooperative interaction'?

5. p6, ‘The shorter dwell times of phosphorylated tau molecules within tau envelopes suggests that phosphorylation impairs the cooperative interaction between phosphorylated and non-phosphorylated tau proteins within the envelopes, consistent with a previous study which found that Cdk5 phosphorylation can induce disassembly of envelopes formed by nonphosphorylated bacterial tau58.’ Is this reference correct? I do not find Cdk5 in this paper.

6. What is ‘SiR-tubulin assay'?

Minor points:

1. p6, ‘Interestingly, we found that HEK tau exhibited the shortest dwell times within the envelopes, ~ 2.3 times shorter than E. coli tau. We speculate this may be attributed to the additional phosphorylation sites (T373 and T377) in the pseudo-repeat Region (R') of HEK tau (Figure 1C)), which has been reported to regulate the cooperative interaction between tau molecules on microtubules13.’ This argument is difficult to follow. Please explain why authors speculate T373 and T377 regulate the cooperative binding.
2. Fig. 1A, ‘N-terminal domain’ should be ‘N-terminal insertions’
3. Fig. 1B, T235 should be S235, T237 should be S237
4. p4. ‘Notably, phosphorylation at S210, S237, S258, and S422 has been only detected in AD patient brains (Figure 1C)59-65.’ I do not think these sites are widely accepted as AD-related phosphorylation sites.

Reviewer #2

(Remarks to the Author)
The submitted study investigates how distinct tau phosphorylation profiles influence key biochemical interactions, including microtubule binding, envelope formation, and oligomerization. The authors employed recombinant tau proteins derived from various expression systems (E. coli, SF9, and HEK cells).

Overall, the manuscript is well written, and the data presented support most of the authors’ conclusions. The study is scientifically relevant, as the role of tau phosphorylation and its functional implications remain incompletely understood.
The data and conclusions are not entirely novel, as phosphorylation has previously been shown to influence tau’s affinity for microtubules and promote oligomerization. The most original aspect of this study lies in the demonstration of how phosphorylation affects envelope formation and microtubule compaction.

Several questions and comments need to be addressed to fully support the main findings of the paper:

1) In Figure 1 the authors report the differential phosphorylation pattern from HEK and SF9 tau measured by MS. In the methods section, the authors cite a couple of methodological references on the approach. At this step, it would be important to clarify how site-specific phosphorylation pattern was obtained, as opposed to peptide-specific pattern. One tryptic peptide may contain several phospho-sites. It is key to describe how single residue resolution is achieved. In addition, the authors do not mention other potential post translational modifications (ie in HEK cells) that may interfere with the analysis.
Figure 1D shows a shift in the SEC profile. However, since tau is tagged with GFP, which may significantly influence the protein’s size and conformation, it is important to repeat this experiment using untagged tau to more robustly support the authors’ conclusions.

2) In Figures 2 and 3, including additional controls, such as phosphatase-treated SF9 tau, would strengthen the conclusions. This would help demonstrate that the observed differences are specifically due to phosphorylation patterns, rather than other uncontrolled variables or potential biases.

3) Figure 4 presents distinct oligomerization profiles. However, it is unclear why GFP-tagged tau was used for the mass photometry analysis. The use of untagged tau would provide more convincing evidence and help rule out potential contributions of the GFP tag, which could itself be differentially modified or influence multimerization independently of tau.

Minor comments:
Given that the study relies on recombinantly produced tau proteins and includes quantitative analyses, it is important to provide uncropped images of SDS-PAGE gels or Coomassie-stained gels for all protein preparations. This would help demonstrate the purity of the samples, confirm the absence of contaminants, and validate the use of equivalent protein concentrations across conditions.

Reviewer #3

(Remarks to the Author)
The present report is a very comprenhensive study and characterization of the tau purified from HEK cells and insect cells in comparison with E coli non phosphorylated tau. The concept that taus with different phosphorylation patterns have different properties it is not novel, but it is important to reinforce it. The tau forms used in the present paper are characterized in vitro after being purified, And the phosphorylation sites are occupied in most cases up to 15%. The properties are determined against taxol stabilized microtubules, which is not a physiological measure. Several concerns has to be addressed: Tau is

only detected by staining and no antibodies, a western blot showing the full length and the degradation products, which can also modified tau behavior. The SDS PAGE shown goes from 75-100, but already tau fragments can be seen.
Whether the differential activity of tau is generated just by different phosphorylation patterns it can be verify by dephosphorylating the different taus and getting similar results.
In Fig 5 most of the SNAP tau disappears after 90 hours incubation, is it possible that the aggregates of dGAC carry on the small concentration aggregates of SNAPtau?
How these forms of phosphorylated tau can be compared with the pathological tau when in the latter tau is almost 100% phosphorylated at positions 198/199/202 and 262 and that is not the case in these tau forms?

Version 1:

Reviewer comments:

Reviewer #1

(Remarks to the Author)
The authors have adequately addressed the reviewers' comments #1 and #2, and the manuscript has been significantly improved. I suggest the authors address the following minor points before it is accepted for publication.
Minor points:
1. Lines 1-2 of the third paragraph on Page 4 and line 7 of the last paragraph of the Results on page 10; “sites” would be “phosphates”.
2. Legend of Fig. 1A right; Please add ‘coomassie-staining’ in the description of SDS-PAGE as Fig. 5B legend.
3. Supplemental Figure S1: Please include higher molecular weight areas of SDS-PAGE to show un-cleaved GFP-tau to show any uncleaved GFP-tau remains.
4. Fig. 5B and C: Please indicate which bands were quantified in the figure legend. SDS-PAGE (B) shows that bands about <100 kDa, which must be the full-length SNAP-tau, peak at 24-48 hr and decrease thereafter; however, the graph (C) shows they plateau after 50h. Is it because the smear bands above 100kDa were included in the measurement?

Reviewer #3

(Remarks to the Author)
This reviewer finds that the authors really addressed the critiques. The only one not addressed is that the binding of tau to microtubules is not really the physiological tau activity, the induction of tubulin assembly into microtubules would be a better physiological measure. May be something could be added to the discussion, because the findings reposted do represent an interesting finding.

Open Access This Peer Review File is licensed under a Creative Commons Attribution 4.0 International License, which permits use, sharing, adaptation, distribution and reproduction in any medium or format, as long as you give appropriate credit to the original author(s) and the source, provide a link to the Creative Commons license, and indicate if changes were made.
In cases where reviewers are anonymous, credit should be given to 'Anonymous Referee' and the source.

The images or other third party material in this Peer Review File are included in the article's Creative Commons license, unless indicated otherwise in a credit line to the material. If material is not included in the article's Creative Commons license and your intended use is not permitted by statutory regulation or exceeds the permitted use, you will need to obtain permission directly from the copyright holder.
To view a copy of this license, visit https://creativecommons.org/licenses/by/4.0/

Author's Rebuttal Letter

Your manuscript entitled "A pathological phosphorylation pattern enhances tau cooperativity on microtubules and facilitates tau filament assembly" has now been seen by 3 referees, whose comments are appended below. You will see from their comments copied below that while they find your work of potential interest, they have raised concerns that must be addressed. In light of these comments, we cannot accept the manuscript for publication, but would be interested in considering a revised version that addresses these concerns.

We hope you will find the referees' comments useful as you decide how to proceed. Should further experimental data or analysis allow you to address these criticisms, we would be happy to look at a substantially revised manuscript. In particular, please consider the referees' requests for testing of other PTM modifications, include phosphatase treatments to confirm the implication of protein phosphorylation, improve characterization of your tools (phosphorylation pattern, effect of GFP-tag), be more explicit in the description of your methodology and more cautious in your interpretation. Please note that we'd need full length gels as Suppl Figs for the final version, too.

We are committed to providing a fair and constructive peer-review process. Do not hesitate to contact us if you wish to discuss the revision or if there are specific requests from the reviewers that you believe are technically impossible or unlikely to yield a meaningful outcome.

Reviewers' comments:

Reviewer #1 (Remarks to the Author):

Fan et al. set out to analyze the properties of tau proteins with different phosphorylation patterns, focusing on microtubule binding, cooperative protein envelope formation, and NFT filament assembly. Tau proteins were expressed in bacteria, Sf9 cells, or HEK293 cells. Bacterial tau proteins are not phosphorylated, and Sf9-tau and HEK293-tau contain more than 50 phosphorylation sites. Phosphorylation at most of those sites was lower than 15%, and 11 or 12 sites had more than 15% of phosphorylation occupancy. They found that Sf9-tau and HEK293-tau show less binding rate to microtubules and to the envelopes than bacterial tau. Sf9-tau and HEK293-tau form oligomers and co-assembled dGAE-formed fibrils more efficiently than bacterial tau. The authors argue that these results reveal new insights into how tau phosphorylation impacts tau's physiological roles on microtubules and its pathoconversion into NFTs.

Their findings are potentially interesting. However, their conclusions are not fully supported by their results. The tau proteins they used are heterogeneous, and the roles of

phosphorylation in their different behavior are not clear. Methods and experimental conditions are not sufficiently described. Results should be interpreted more carefully without bias. Many more experiments would be needed to support their conclusions.

Major points:

1. Tau proteins from cells are heterogeneous. As they showed, Sf9 tau proteins have about 5-6 phosphorylated sites per molecule and 10% of the average phosphorylation degree (it is not clear whether this means 10% of tau proteins are phosphorylated, or 10% of Ser, Thr, or Tyr in a tau protein are phosphorylated), thus, they are a mixture of tau proteins with different phosphorylation patterns. Authors argue that Sf9 tau has a pathological phosphorylation pattern since phosphorylated sites in Sf9 tau include several AD-related sites (see minor comment 1); however, to analyze the roles of tau phosphorylation at AD-related sites, it would be more straightforward to use tau proteins with specific phosphorylation pattern. Further attempts to purify phosphorylated tau, such as in vitro phosphorylation or immunoprecipitation or site-specific mutagenesis of specific phosphorylation sites, would be required to test the roles of phosphorylation.

We thank the reviewer for their insightful comments and the opportunity to clarify these important points. We will address each concern below and have revised the manuscript accordingly.

1. Clarification of Phosphorylation Stoichiometry:

We apologize for the lack of clarity regarding the phosphorylation quantification. The reviewer is correct to question the "10%" phrasing.

To clarify, our mass spectrometry data indicate an average phosphorylation stoichiometry of ~5-6 phosphate groups per tau molecule. This was calculated by quantifying the occupancy (the fraction of the total protein pool that is phosphorylated at a given site) for each of the 58 phosphorylated sites we identified and then summing these occupancy values. An average occupancy of ~10% across these 58 sites corresponds to a total of ~5.8 phosphate groups per molecule.

We have now revised the text in the Results section to remove the ambiguous percentage and state this stoichiometry directly and clearly.

2. On the Use of a Heterogeneous, Cell-Derived Phospho-Tau Model:

We agree with the reviewer that our Sf9-expressed tau is a heterogeneous mixture of phospho-isoforms. However, we respectfully argue that this heterogeneity is a key feature that enhances the **physiological relevance** of our model system, for two main reasons:

1. **Enrichment for Pathological Sites:** As the reviewer notes, our analysis confirmed that this mixture is reproducibly enriched for several key AD-related phosphorylation sites. This provides a valuable tool for studying the collective, synergistic effects of multiple pathological phosphorylations, which cannot be captured by analyzing single sites in isolation.
2. **Reflecting In Vivo Complexity:** Tau phosphorylation in the brains of AD patients is also known to be highly heterogeneous, with significant variability in phospho-sites among individuals (e.g., PMID: 33188775). Therefore, while systems with single, specific phosphorylation sites are valuable for dissecting individual molecular events, and have been extensively utilized in the field, our model allows us to study the functional consequences of a pathologically relevant **ensemble** of phosphorylations, which may better reflect the complex in vivo state, and makes our approach more unique in the field.

We have added a sentence to the Discussion to better frame the strengths of our approach in this context.

3. Regarding Future Directions (Site-Specific Mutagenesis):

We thank the reviewer for suggesting several excellent follow-up approaches, such as site-specific mutagenesis and in vitro phosphorylation. We agree that these experiments would provide complementary insights into the role of specific phosphorylation sites in the mechanisms we have uncovered.

However, given the substantial effort required to generate and analyze a comprehensive set of phospho-mutants, we believe these studies constitute a distinct and exciting future research direction that builds directly upon the foundational findings of this manuscript.

We have added a sentence to the Discussion section to explicitly acknowledge these important future directions and better position our current findings.

2. Tau proteins from cells have various post-translational modifications. The difference in post-translational modifications other than phosphorylation may contribute to the different behaviors of Sf9-tau and HEK-tau. Mass Spectrometry would have detected post-translational modifications; however, authors seem to ignore PTM other than phosphorylation. They should include such data and compare all post-translational modifications between Sf9 and HEK293.

We thank the reviewer for this useful suggestion. We agree that other post-translational modifications present on eukaryotic cell-expressed tau could in principle influence its behavior, and we appreciate the opportunity to address this directly.

To confirm that the differences we observe are driven primarily by phosphorylation, we have now measured full binding curves for Sf9 tau and LPP-treated Sf9 tau (Figure S2E).

Dephosphorylation substantially reduced the enhanced cooperativity of Sf9 tau, as reflected by a decrease in the Hill coefficient from 2.88 to 1.95, and markedly increased its microtubule affinity, reducing the apparent K_d from ~54 nM to ~10 nM. While these values do not fully recapitulate those of non-phosphorylated *E. coli* tau (h = 1.24, K_d = 6.19 nM), they move substantially in that direction, consistent with either incomplete dephosphorylation by LPP or a minor residual contribution from other post-translational modifications. Importantly, at the single-molecule level, LPP treatment restored both the dwell time (~1.59 seconds to ~4.42 seconds) and landing rate of Sf9 tau to values closely comparable to those of *E. coli* tau (Figure S2A-D), further supporting the conclusion that phosphorylation is the primary driver of the altered microtubule-binding dynamics we observe.

We were unable to measure the binding curves of LPP-treated HEK tau due to its very low yield. As noted in the manuscript, HEK tau required several months to accumulate sufficient material across three independent preparation batches. We note, however, that the physiological-like phosphorylation pattern of HEK tau has comparatively modest effects on tau cooperativity and tau filament assembly relative to Sf9 tau, making the LPP rescue experiment less critical for this variant to support our main conclusions. We have added the new LPP binding curve data to the supplementary materials and updated the Results section accordingly (Figure S2).

3. Authors should describe the experimental methods and conditions in more detail. 'Tau envelope' is not a commonly used assay and needs explanation. In Figure 2 and Figure 3, different concentrations are used in each experiment for each tau. Are they 'single molecule concentration'? What is it and how were they determined? Concentration would affect Hill co-efficient and Kd. Is it okay to compare them from these experiments and conclude they are ‘very similar’?

We thank the reviewer for highlighting these areas where our methods and experimental rationale were unclear. We have provided detailed clarifications below and have significantly revised the Methods and figure legends to incorporate this information.

1. Definition of the "Tau Envelope" Assay:

We apologize for not defining this term clearly. The "tau envelope" assay is a fluorescence microscopy-based method we use to visualize and quantify the **cooperative, high-density binding of tau to the microtubule lattice**. At nanomolar concentrations, tau transitions from binding as discrete single molecules to forming a continuous, sheath-like "envelope" along the microtubule. This assay allows us to measure the parameters of this cooperative binding. The formation of tau envelopes has been extensively characterized by our group, and others (PMIDs: 37747123, 35996000, 31481790, 31481789) in the field and we now ensure these foundational studies are clearly defined and cited in the revised manuscript.

Action: We have now added a clear and detailed definition of tau envelopes in the introduction and a description of the "tau envelope" assay to the Results section: "*We and others have proposed that one of the key functions of the tau protein is its ability to form cohesive tau envelopes on the microtubule surface in a concentration-dependent manner*[13, 14, 17, 18, 56]*. These tau envelopes act as a selectively permeable barrier to regulate the activities of other microtubule-associated proteins (MAPs)*[13, 14]*. Within the envelope, tau proteins exhibit cooperative interactions with each other, resulting in longer dwell times compared to tau proteins outside the envelope*[13, 14]*. To investigate whether phosphorylation affects the interaction of tau proteins within these envelopes, we employed a single-molecule assay to measure the dwell times of differentially phosphorylated tau proteins within envelopes formed by non-phosphorylated E. coli tau.*"

2. Clarification of Concentrations Used in Figure 2 vs. Figure 3:

The reviewer is correct that we used different concentration regimes for the experiments in Figure 2 and Figure 3. This was intentional, as the two assays are designed to measure distinct biophysical properties:

- **Figure 2 (Single-Molecule Analysis):** For these experiments, we used **low picomolar concentrations.** This ensures that individual fluorescent tau molecules are spatially resolved on the microtubule, allowing us to accurately measure single-molecule properties like dwell time and diffusion without signal overlap or influence from tau-tau interactions that may occur at higher densities of molecules.
- **Figure 3 (Cooperative Binding / Envelope Formation):** For these experiments, we used a range of nanomolar concentrations. This concentration regime is where tau exhibits cooperative binding and forms envelopes along microtubules. We chose concentrations that could form envelopes with distinct edges on the microtubule, usually in the nanomole range based on previous research (PMID: 31481790, PMID: 31481789, PMID: 35996000). These concentrations were selected because differentially phosphorylated tau proteins could form envelopes of similar sizes at these concentrations.

Action: We have clarified the rationale for the concentration ranges in the relevant Methods sections and figure legends.

3. Validity of Comparing K_d and Hill Coefficient Values:

We understand the reviewer's concern regarding the comparison of K_d and Hill coefficient values derived from these experiments. We respectfully clarify that these parameters were determined in a manner that makes the comparison valid.

The dissociation constant (K_d) and Hill coefficient (n) are <u>intrinsic properties of the binding interaction (under identical experimental conditions), not dependent on a single</u>

experimental concentration. They are determined by titrating a wide range of protein concentrations, measuring the binding response at each concentration, and fitting the resulting saturation curve to the Hill equation.

For each tau variant analyzed in Figure 3, the reported K_d and Hill coefficient values were derived from a full titration curve, where we measured the extent of microtubule binding across multiple tau concentrations spanning the nanomolar range. Therefore, the comparison of these values is valid because each one represents a complete, independent measurement of that variant's binding properties.

Action: We have revised the legend for Figure 3 to explicitly state: "The reported K_d and Hill coefficient for each tau variant were derived by fitting a full binding curve from a titration series of tau concentrations." This will eliminate any ambiguity for the reader.

4. Figure 3. Within the envelope, a tau protein would interact with other tau proteins and microtubules. Which interaction does the measurement reflect? Authors state that 'Furthermore, the dissociation constant (K_d) values of E. coli tau, HEK tau, and Sf9 tau were 6.19 nM,17.64 nM, and 59.71 nM respectively (Figure 3B), confirming that phosphorylation decreases the microtubule affinity of tau protein, consistent with our single-molecule results (Figure 2).; however, I thought 3B calculate interaction between HEK tau and envelope tau. What is 'cooperative interaction'?

We thank the reviewer for this important question, which highlights an area where our explanation of the assay and its interpretation was not sufficiently clear. We apologize for the confusion and offer the following clarification, which we have incorporated into the revised manuscript.

1. Defining the Interactions Measured and "Cooperative Interaction":

The reviewer is correct that within a tau "envelope," both tau-microtubule and tau-tau interactions are occurring. Our assay is designed to measure the overall process of tau accumulating on the microtubule lattice, and we use the Hill equation to deconvolve the two key thermodynamic contributions to this process:

- **Intrinsic Tau-Microtubule Affinity:** This is the inherent binding strength of a single tau molecule to the microtubule surface.
- **Cooperative Interaction:** This refers to the favorable interactions between adjacent tau molecules already bound to the microtubule. In simple terms, the binding of one tau molecule makes it energetically easier for subsequent tau molecules to bind nearby. This is what drives the transition from diffuse single-molecule binding at low concentrations to the formation of a dense, stable "envelope" at higher concentrations.

We use two parameters from the Hill plot to quantify these effects:

- The **K_d (apparent dissociation constant)** primarily reflects the intrinsic tau-microtubule affinity. It is the concentration at which half-maximal binding is achieved.
- The **Hill coefficient (h)** specifically quantifies the degree of cooperativity. A value of $n > 1$ indicates positive cooperativity, confirming that tau-tau interactions facilitate further binding.

2. Clarifying the Experimental Design (Titration vs. Single Point):

We would like to clarify a key misunderstanding, which we regret was not made clear in the original text. The data in Figure 3B are not from a single experiment where we measure, for example, "interaction between HEK tau and envelope tau."

Instead, the binding curve for each tau variant is generated from a full titration series. We perform multiple experiments, each with a different concentration of a single tau variant (e.g., HEK tau), and measure the amount bound to the microtubule at equilibrium. By plotting the amount bound versus concentration, we generate the full saturation curve.

Therefore, the assay measures the process of a given tau variant building up its *own* envelope on the microtubule lattice.

3. Justification for the Conclusion:

With this clarification, we believe our original conclusion is robust. By comparing the K_d values derived from these full binding curves, we can directly assess how phosphorylation impacts the overall affinity of tau for the microtubule. The observed increase in K_d with phosphorylation (*E. coli* < HEK < Sf9) demonstrates a decrease in binding affinity. This finding is fully consistent with our independent single-molecule dwell time measurements in Figure 2, strengthening our overall conclusion.

Action: We have substantially revised the Results section and the legend for Figure 3. We now explicitly define "cooperative interaction" and clarify that the reported Kd and Hill values are derived from fitting full titration curves. We believe these revisions will resolve the reviewer's confusion.

5. p6, 'The shorter dwell times of phosphorylated tau molecules within tau envelopes suggests that phosphorylation impairs the cooperative interaction between phosphorylated and non-phosphorylated tau proteins within the envelopes, consistent with a previous study which found that Cdk5 phosphorylation can induce disassembly of envelopes formed by nonphosphorylated bacterial tau58.' Is this reference correct? I do not find Cdk5 in this paper.

We apologize for the confusion! This sentence should be reference citation number 56. This paper has now been published (PMID: 41593416). We have updated the citation in the revised manuscript. Figure 1e-g and Figure 2f-h in the cited paper respectively demonstrate that phosphorylation of bacterial tau by Cdk5 induce envelope disassembly and reduces envelope integrity respectively. We regret the oversight and have thoroughly reviewed our citation, updating it in this revised version.

6. What is 'SiR-tubulin assay'?

Sorry for the confusion, we have included a brief description of the 'SiR-tubulin assay' in the revised manuscript and a citation to its previous use in our work.

Minor points:

1. p6, 'Interestingly, we found that HEK tau exhibited the shortest dwell times within the envelopes, ~ 2.3 times shorter than E. coli tau. We speculate this may be attributed to the additional phosphorylation sites (T373 and T377) in the pseudo-repeat Region (R') of HEK tau (Figure 1C), which has been reported to regulate the cooperative interaction between tau molecules on microtubules13.' This argument is difficult to follow. Please explain why authors speculate T373 and T377 regulate the cooperative binding.

We thank the reviewer for highlighting this point and for giving us the opportunity to better explain our reasoning. We apologize that the logic behind our speculation was not sufficiently clear.

Our speculation is built on two key findings—one from our previous work and one from the current study:

1. **Established Function of R':** Our previous research established that the pseudo-repeat region (R') is a primary mediator of the lateral, cooperative tau-tau interactions that stabilize the tau envelope (Tan et al., 2019; PMID: 31481790).
2. **Current Observation:** In this study, we observe that HEK tau exhibits the most significantly reduced dwell time within an envelope, which indicates that its cooperative interactions are the most impaired among the variants. When we examined the unique features of the HEK tau phosphoproteome, we noted phosphorylation at sites T373 and T377, which are located directly within the critical R' region.

Hypothesis:
Based on these points, we hypothesize that the introduction of negatively charged phosphate groups directly into the R' region—the very domain responsible for mediating favorable tau-tau contacts—would interfere with its function. This interference could be

due to electrostatic repulsion or steric hindrance, which would disrupt the lateral interactions between adjacent tau molecules. This disruption of the R'-mediated cooperative network would lead directly to the weakened binding and shorter dwell times we observe for HEK tau within the envelope.

Action: We have revised the text in the Results section to incorporate this explicit reasoning, making the link between the phosphorylation sites and the functional outcome clear. *"We speculate that this is because phosphorylation at T373, and to a lesser extent T377, both of which lie within the pseudo-repeat region R' and are HEK-specific modifications, interferes with the lateral tau-tau cooperative interactions that R' is known to mediate[13] (Figure 1C, Supplementary Table 1). T373 in particular, phosphorylated at ~30% occupancy, would introduce a negatively charged phosphate group directly into this critical domain, potentially disrupting favorable tau-tau contacts via electrostatic repulsion or steric hindrance.."*

2. Fig. 1A, 'N-terminal domain' should be 'N-terminal insertions'

We thank the reviewer for pointing out the mistake and we have now updated it.

3. Fig. 1B, T235 should be S235, T237 should be S237

We thank the reviewer for pointing out the mistake and we have now updated it.

4. p4. 'Notably, phosphorylation at S210, S237, S258, and S422 has been only detected in AD patient brains (Figure 1C) 59-65.' I do not think these sites are widely accepted as AD-related phosphorylation sites.

We thank the reviewer for this important critique. The reviewer is correct that our original phrasing, 'only detected in AD patient brains,' was an overstatement, and we apologize for this imprecision. We have substantially revised the manuscript to frame our findings more accurately, and now state that these sites are 'prominently featured within the complex phosphoproteome of AD brains.' We also wish to correct an error in our original response: we previously included pS258 in the list of AD-relevant sites present in Sf9 tau. Upon careful re-examination of our mass spectrometry data, S258 phosphorylation occupancy is 0% in Sf9 tau and is in fact a HEK-specific modification (Supplementary Table 1). We apologize for this oversight and have corrected the manuscript accordingly, removing S258 from the list of AD-relevant Sf9 tau sites. The three Sf9-specific sites we highlight are supported as follows:

We wish to briefly justify our focus on this specific group of sites, as they represent different facets of the disease process and are supported by strong evidence in the literature:

- **pS422:** This is an established marker of mature tau pathology. Its phosphorylation is strongly correlated with the presence of neurofibrillary tangles (NFTs) and the severity of neuronal cytopathology in Alzheimer's disease (Augustinack et al., *Acta Neuropathol*, 2002; PMID: 11837744).
- **pS210:** This site has been directly identified by mass spectrometry in paired helical filament (PHF)-tau isolated from AD brains and is considered a key site for the action of multiple kinases implicated in AD, such as GSK-3β and CDK5 (Hanger et al., *J Biol Chem*, 2007; PMID: 17562708).
- **pS237:** While not a canonical standalone marker, this site is identified in modern, large-scale phosphoproteomic analyses that map the extensive heterogeneity of tau modifications in post-mortem AD brain tissue (Wesseling et al., *Cell*, 2020; PMID: 33188775).

Therefore, one of the primary goals of our study is to understand the functional consequences of a phosphorylation pattern that includes sites associated with different stages of AD pathology. We believe this approach provides a more holistic view of how specific patterns of phosphorylation, rather than just individual sites, drive tauopathy.

Action: We have revised the sentence on Page 4 to be more accurate and have ensured the Discussion appropriately frames the significance of studying this collective, pathologically relevant pattern.

Reviewer #2 (Remarks to the Author):

The submitted study investigates how distinct tau phosphorylation profiles influence key biochemical interactions, including microtubule binding, envelope formation, and oligomerization. The authors employed recombinant tau proteins derived from various expression systems (E. coli, SF9, and HEK cells).

Overall, the manuscript is well written, and the data presented support most of the authors' conclusions. The study is scientifically relevant, as the role of tau phosphorylation and its functional implications remain incompletely understood.

The data and conclusions are not entirely novel, as phosphorylation has previously been shown to influence tau's affinity for microtubules and promote oligomerization. The most original aspect of this study lies in the demonstration of how phosphorylation affects envelope formation and microtubule compaction.

Several questions and comments need to be addressed to fully support the main findings of the paper:

1) In Figure 1 the authors report the differential phosphorylation pattern from HEK and SF9 tau measured by MS. In the methods section, the authors cite a couple of methodological references on the approach. At this step, it would be important to clarify how site-specific phosphorylation pattern was obtained, as opposed to peptide-specific pattern. One tryptic peptide may contain several phospho-sites. It is key to describe how single residue resolution is achieved. In addition, the authors do not mention other potential post translational modifications (ie in HEK cells) that may interfere with the analysis. Figure 1D shows a shift in the SEC profile. However, since tau is tagged with GFP, which may significantly influence the protein's size and conformation, it is important to repeat this experiment using untagged tau to more robustly support the authors' conclusions.

We thank the reviewer for highlighting the need to clarify how site-specific phosphorylation was determined.

In our analysis, phosphorylation site localization was performed using ProteinLynx Global Server (PLGS), which is designed to identify variable post-translational modifications, including phosphorylation on serine (S), threonine (T), and tyrosine (Y) residues.

For peptides containing potential multiple phosphorylation sites, PLGS achieves single-residue resolution through fragment ion analysis. Specifically, site localization is based on the detection of diagnostic fragment ions (b- and/or y-ions) that unambiguously assign the phosphate group to a particular residue within the peptide sequence. When fragment ions spanning a given residue are observed in both modified and unmodified forms, PLGS can confidently localize the phosphorylation site.

PLGS uses a probabilistic scoring framework that incorporates:

- The match between observed and theoretical fragment ions
- The presence and intensity of site-determining ions (diagnostic b/y ions)
- The overall peptide score, which reflects the confidence of peptide identification
- A modification localization score, which ranks possible phosphorylation sites within a peptide based on the best-supported fragmentation pattern

Only phosphorylation sites supported by high-confidence fragment ion evidence and meeting PLGS scoring thresholds were considered in this study.

Regarding other post-translational modifications, the database search included common variable modifications (e.g., oxidation of methionine). We acknowledge that additional PTMs present in HEK-expressed tau could, in principle, influence peptide identification; however, the stringent scoring criteria and requirement for diagnostic fragment ions minimize ambiguity in phosphorylation site assignment.

Action: We have added a sentence to the Discussion to acknowledge this point: *"While we recognize that other post-translational modifications are potentially present on our cell-derived tau, our study was designed to specifically isolate and characterize the functional consequences of the distinct phosphorylation patterns, which are central to tau's regulation and pathology."* In addition, we have now clarified these points in the Methods section.

2. On the SEC Profile and the Influence of the sfGFP Tag:

The reviewer identifies the N-terminal sfGFP tag as a potential confounding factor in interpreting the SEC data in Figure 1D. We agree that a tag of this size can influence the hydrodynamic radius of a fusion protein, and thank the reviewer for pushing us to address this directly.

To verify that the earlier elution of Sf9 tau reflects a property of the tau molecule itself, we repeated the SEC experiment following proteolytic removal of the sfGFP tag for *E. coli* tau and Sf9 tau, and have included this data in the revised supplementary materials (Figure S1). The earlier elution of tag-free Sf9 tau relative to tag-free *E. coli* tau was preserved, confirming that the subtle shift is not an artifact of the GFP fusion. We were unfortunately unable to generate sufficient quantities of tag-free HEK tau for this comparison due to the low yield of this preparation, and we now explicitly acknowledge this limitation in the revised manuscript, noting that a potential tag contribution to the HEK tau elution profile cannot be formally excluded.

Regardless, the primary purpose of the SEC experiment is to confirm that all three preparations are composed of largely homogenous, non-aggregated populations under these conditions, which it clearly demonstrates. Our paper's major conclusions do not rest on the subtle Sf9 tau shift. The more definitive evidence for functional differences between variants comes from orthogonal biophysical measurements where tag influence is minimal or absent: the dramatically different Hill coefficients in the cooperative binding assay (Figure 3B), the distinct oligomerization profiles measured by mass photometry (Figure 4), and the markedly different fibrillization kinetics (Figure 5C).

Action: We have revised the Results section to tone down our interpretation of the SEC data. The revised text now reads:

"The chromatographic profiles revealed that HEK tau eluted at a highly similar elution volume to E. coli tau, whereas Sf9 tau eluted slightly earlier than both E. coli tau and HEK tau (Figure 1D). This subtle shift in Sf9 tau could suggest a modest conformational change in this phosphorylation variant. To assess whether the large N-terminal sfGFP tag might contribute to this result, we repeated the SEC analysis with E. coli tau and Sf9 tau following proteolytic removal of the tag (Figure S1). The earlier elution of tag-free Sf9 tau relative to tag-free E. coli tau was preserved, confirming that this shift reflects a property of the tau molecule itself rather than an artifact of the sfGFP fusion. We were unable to generate

sufficient quantities of tag-free HEK tau for this comparison, and therefore interpret the HEK tau elution profile with the caveat that a potential tag contribution cannot be formally excluded. Nonetheless, all three preparations eluted as largely homogenous populations under these conditions, and more definitive evidence for functional differences between these variants is provided by subsequent assays".

2) In Figures 2 and 3, including additional controls, such as phosphatase-treated SF9 tau, would strengthen the conclusions. This would help demonstrate that the observed differences are specifically due to phosphorylation patterns, rather than other uncontrolled variables or potential biases.

We thank the reviewer for these helpful suggestions. We have measured the dwell time and landing rate of phosphatase treated Sf9 tau. These new results show that dephosphorylation significantly increased both the dwell time (~1.59 seconds to ~4.42 seconds) and landing rate of Sf9 tau on microtubules (Figure S2A-D), restoring values to a range comparable to those of non-phosphorylated *E. coli* tau (Figure 2B, C). Additionally, dephosphorylation substantially reduced the enhanced cooperativity of Sf9 tau, as reflected by a decrease in the Hill coefficient from 2.88 to 1.95 and a reduction in Kd from ~54 nM to ~10 nM (Figure S2E), although these values do not fully converge on those of *E. coli* tau, possibly reflecting incomplete dephosphorylation.

3) Figure 4 presents distinct oligomerization profiles. However, it is unclear why GFP-tagged tau was used for the mass photometry analysis. The use of untagged tau would provide more convincing evidence and help rule out potential contributions of the GFP tag, which could itself be differentially modified or influence multimerization independently of tau.

We thank the reviewer for the helpful suggestions. The size of the untagged full-length tau monomer (approximately 46 kDa for the 2N4R isoform) is very close to the lower limit of reliable mass detection for our mass photometer, making it technically challenging and unreliable to repeat this assay using untagged protein. For this reason, and to ensure confident detection of monomeric and oligomeric species, we used N-terminally sfGFP-tagged tau, which shifts the expected monomer mass well into the robust detection range of the instrument.

Importantly, sfGFP itself has been extensively engineered and characterized as a robustly folded, soluble fluorescent protein that maintains its folding properties even when fused to aggregation-prone partners. The original description of sfGFP (Pédelacq et al., Nat Biotechnol 2006, PMID: 16369541) showed that sfGFP tolerates circular permutations, resists chemical denaturation, and folds efficiently when fused to poorly folded polypeptides, indicating that the GFP moiety is unlikely to drive nonspecific aggregation of its fusion partner. Structural and biophysical analyses further demonstrate that sfGFP is predominantly monomeric or at most a weak dimer under typical experimental conditions

(below tens of micromolar), with any self-association being relatively low affinity compared to classical oligomerizing fluorescent proteins. These properties were a key reason for choosing sfGFP rather than other GFP variants, which are more prone to misfolding or oligomerization.

Consistent with these prior characterizations, our mass photometry measurements of sfGFP–tau detected discrete tau-containing species at masses substantially larger than sfGFP alone, and the observed oligomerization profiles differed between the tau variants while using the identical sfGFP tag. This argues that the distinct species we observe arise from tau–tau interactions rather than sfGFP-driven multimerization. We have clarified this rationale and now explicitly cite the original sfGFP characterization in the revised manuscript.

We agree that removing the GFP-tag will provide more convincing evidence, and may rule out potential contributions of the tag, while our goal in this article is to investigate the effect of different phosphorylation patterns on the tau's microtubule binding, cooperative interactions on the microtubule, and PHF-like fibrils assembly, from molecular level, using TIRFM. The fluorescent tag or dye labeled tag is critical for the microscopy-based in vitro study. Additionally, there are a couple of studies that used GFP-tagged tau to investigate its oligomeric states both in vitro and in cells, and no tag induced dimerization or other effects have been reported (PMID: 29472250, PMID: 33952699, PMID: 32499559, PMID: 41610849). Finally, in our updated work, we removed the GFP tag by cleavage of PreScission Protease, it's gel filtration profile show that there wasn't a significant change after the cleavage (Figure S1). Therefore, we hope our data is sufficient to address the reviewer's concern.

Minor comments:

Given that the study relies on recombinantly produced tau proteins and includes quantitative analyses, it is important to provide uncropped images of SDS-PAGE gels or Coomassie-stained gels for all protein preparations. This would help demonstrate the purity of the samples, confirm the absence of contaminants, and validate the use of equivalent protein concentrations across conditions.

We thank the reviewer for these valuable suggestions! We have included intact PAGE gels for all protein preparations and concentration validations in the supplementary data of our revised manuscript (Figure S3-S8).

Reviewer #3 (Remarks to the Author):

The present report is a very comprenhensive study and characterization of the tau purified from HEK cells and insect cells in comparison with E coli non phosphorylated tau. The concept that taus with different phosphorylation patterns have different properties it is not novel, but it is important to reinforce it. The tau forms used in the present paper are characterized in vitro after being purified, And the phosphorylation sites are occupied in most cases up to 15%. The properties are determined against taxol stabilized microtubules, which is not a physiological measure. Several concerns has to be addressed: Tau is only detected by staining and no antibodies, a western blot showing the full length and the degradation products, which can also modified tau behavior. The SDS PAGE shown goes from 75-100, but already tau fragments can be seen. Whether the differential activity of tau is generated just by different phosphorylation patterns it can be verify by dephosphorylating the different taus and getting similar results.

In Fig 5 most of the SNAP tau disappears after 90 hours incubation, is it possible that the aggregates of dGAC carry on the small concentration aggregates of SNAPtau? How these forms of phosphorylated tau can be compared with the pathological tau when in the latter tau is almost 100% phosphorylated at positions 198/199/202 and 262 and that is not the case in these tau forms?

We sincerely thank the reviewer for their comprehensive feedback and critical suggestions. These comments have helped us to significantly clarify the manuscript and strengthen our conclusions. We will address each of the reviewer's points in order.

1. On Protein Purity and Integrity:

The reviewer raised a valid concern about the purity and integrity of our tau preparations.

- **Purification:** As the reviewer noted, our three-step purification protocol (affinity, ion-exchange, and size-exclusion chromatography) is designed to yield highly pure protein. The symmetrical peaks from our final gel filtration step (Figure 1D) are indicative of homogeneous, non-aggregated protein preparations. Further, our mass photometery data (Figure 4) also reveals the expected size for our tau preparations with no major contaminants of other molecular mass. This technique has single molecule sensitivity and is therefore at the extreme end of sensitivity for detecting molecular species, range from ~30 kDa to 5 MDa (PMID: 38664367).
- **Integrity:** To directly address the concern about potential degradation products, we have now included the full, uncropped Coomassie-stained SDS-PAGE gels for all three final purified tau preparations in the supplementary materials **(Figure S7)**. These gels show a prominent, sharp band at the expected molecular weight for full-length sfGFP-tau, with minimal evidence of lower molecular weight degradation products.

2. On the Dephosphorylation Control Experiment:

The reviewer proposes an excellent experiment: dephosphorylating HEK and Sf9 tau to see if their properties revert.

We thank the reviewer for this important suggestion. To directly test whether the differential behaviors we observe arise from phosphorylation rather than other post-translational modifications, we have now performed LPP dephosphorylation of Sf9 tau and included these results in the revised supplementary materials (Figure S2).

At the single-molecule level, LPP treatment restored both the dwell time (~1.59 seconds to ~4.42 seconds) and the landing rate of Sf9 tau to values closely comparable to those of non-phosphorylated *E. coli* tau (Figure S2A-D), directly confirming that phosphorylation is the primary driver of the altered microtubule-binding dynamics we observe for Sf9 tau. At the ensemble level, dephosphorylation substantially reduced the enhanced cooperativity of Sf9 tau, reflected by a decrease in the Hill coefficient from 2.88 to 1.95 and a reduction in the apparent Kd from ~ 54 nM to ~10 nM (Figure S2E). While these values do not fully converge on those of non-phosphorylated *E. coli* tau (h = 1.24, Kd = 6.19 nM), they move substantially in that direction, consistent with either incomplete dephosphorylation by LPP or a minor residual contribution from other post-translational modifications present on Sf9-expressed tau. Taken together, these results confirm that phosphorylation is the primary determinant of the enhanced cooperativity and reduced microtubule affinity of Sf9 tau and are consistent with prior literature on the effects of phosphorylation on overall microtubule affinity.

We were unable to perform the equivalent LPP rescue experiment for HEK tau for two reasons. First, the yield of HEK tau is low and required substantial effort across three independent preparation batches to accumulate sufficient material for the experiments already reported in the manuscript. Generating additional LPP-treated HEK tau at the scale required for full binding curves was not feasible for us. Second, and more importantly, the biological motivation for this control is less compelling for HEK tau than for Sf9 tau. Our data show that the physiological-like phosphorylation pattern of HEK tau does not dramatically alter cooperative interactions between tau molecules during envelope formation, and HEK tau does not show enhanced co-assembly into NFT-like filaments. The functional differences that most require a phosphorylation-specific explanation are those observed for Sf9 tau, and the LPP rescue experiment presented here directly addresses that question. We have updated the Results section to reflect these new findings and hope this satisfactorily addresses the reviewer's concern.

3. On the Mechanism of Co-assembly in Figure 5:

The reviewer questions whether the SNAP-tau is truly incorporating into the dGAE filaments or simply being passively "carried on" with the pellet.

This is a critical point, and our data strongly support a specific incorporation mechanism. In our control experiments, SNAP-tau alone does not form aggregates or pellet upon ultracentrifugation under these conditions (see "Tau (*E. coli*)", "Tau (HEK)", and "Tau (Sf9)" curves in Figure 5C). It only appears in the pellet fraction in the presence of dGAE filaments.

Furthermore, if the mechanism were simple, non-specific co-precipitation, one would expect similar kinetics for all tau variants. Instead, we observe dramatically different incorporation kinetics ($t_{1/2}$), with the Sf9 tau (pathological-like pattern) incorporating significantly faster than the others. This difference in kinetics strongly implies that the specific phosphorylation pattern of SNAP-tau is actively influencing its ability to engage with and incorporate into the growing filament, rather than being passively carried along. We have revised the text to state this rationale more clearly when presenting the co-assembly data.

4. On the Comparison to Pathological Tau and Phosphorylation Occupancy:

This is the most crucial point. The reviewer correctly states that end-stage NFTs in AD brains contain tau with very high phosphorylation occupancy at certain sites.

We agree completely. Our goal with this study is not to perfectly replicate the biochemical state of an end-stage, insoluble NFT. Rather, our aim is to model the earlier, functional consequences of a pathologically-relevant *pattern* of phosphorylation on a soluble, functional tau molecule.

The field is increasingly recognizing that tauopathy is a long-term process and that early, subtle changes in phosphorylation can trigger the conformational shifts and altered interactions that lead to aggregation. Even if the occupancy at any single site is low, the cumulative, synergistic effect of multiple phosphorylations across the protein can have profound functional consequences. Our data powerfully support this concept: even with low average stoichiometry, we observe dramatic changes in microtubule affinity, cooperativity, and filament incorporation propensity.

Our work therefore provides insight into a critical, earlier stage of the disease process: how a specific "pathological pattern" of phosphorylation, even at low levels, primes tau for dysfunction long before it becomes hyperphosphorylated and fully insoluble. We have substantially revised the Discussion to state this rationale explicitly, better framing the relevance and limitations of our model system and highlighting that our key conclusion is about the *power of the pattern*, not the overall level of phosphorylation.

Reviewers' comments:

Reviewer #1 (Remarks to the Author):

The authors have adequately addressed the reviewers' comments #1 and #2, and the manuscript has been significantly improved. I suggest the authors address the following minor points before it is accepted for publication.

Minor points:

1. Lines 1-2 of the third paragraph on Page 4 and line 7 of the last paragraph of the Results on page 10; “sites” would be “phosphates”.

We thank the reviewer for this professional suggestion. We have now updated the descriptions in both sections.

2. Legend of Fig. 1A right; Please add ‘coomassie-staining’ in the description of SDS-PAGE as Fig. 5B legend.

We thank the reviewer for this helpful suggestion. We have updated the description of SDS-PAGE in Fig. 1A.

3. Supplemental Figure S1: Please include higher molecular weight areas of SDS-PAGE to show un-cleaved GFP-tau to show any uncleaved GFP-tau remains.

We thank the reviewer for the important suggestion. We have now included higher molecular weight areas of SDS-PAGE in Supplemental Figure S1.

4. Fig. 5B and C: Please indicate which bands were quantified in the figure legend. SDS-PAGE (B) shows that bands about <100 kDa, which must be the full-length SNAP-tau, peak at 24-48 hr and decrease thereafter; however, the graph (C) shows they plateau after 50h. Is it because the smear bands above 100kDa were included in the measurement?

We thank the reviewer for this critical suggestion. Yes, the smear bands above full-length SNAP-tau proteins were included in the quantification, as we show in our mass photometry data that tau proteins could form small oligomers in solution (Fig. 4). We have now updated the description for full-length SNAP-tau quantification in the legend of Fig. 5B.

Reviewer #3 (Remarks to the Author):

This reviewer finds that the authors really addressed the critiques. The only one not addressed is that the binding of tau to microtubules is not really the physiological tau activity, the induction of tubulin assembly into microtubules would be a better physiological measure. May be something could be added to the discussion, because the findings reposted do represent an interesting finding.

We thank the reviewer for this helpful point. We agree that simple microtubule binding is not, by itself, a complete measure of tau's physiological activity. Our goal here was not to argue that binding alone defines tau function, but to test how different phosphorylation patterns alter microtubule-associated tau behaviors that are likely relevant to physiology and disease. In particular, our data show that although phosphorylation reduces overall microtubule affinity, phosphorylated tau still forms cooperative envelopes on microtubules, and both HEK tau and Sf9 tau retain the ability to compact the microtubule lattice in vitro. Because tau envelopes have been proposed to regulate access of other microtubule-associated proteins to the lattice, these results support the idea that phosphorylated tau can preserve key microtubule-based functions even when its binding affinity is reduced. At the same time, we agree that direct tubulin polymerization or microtubule assembly assays would provide an additional functional readout, and this is an important direction for future work. To address the reviewer's concern, we have revised the Discussion to clarify that our study focuses on microtubule binding, cooperative envelope formation, and lattice compaction as mechanistically informative tau activities, while acknowledging that these assays do not capture the full range of tau's physiological functions.
