# Supplementary material for: A pathological phosphorylation pattern enhances tau cooperativity on microtubules and facilitates tau filament assembly
Source: Commun Biol. 2026 Aug 21;9:1408. doi: 10.1038/s42003-026-10797-6 (PMC13714281; doi:10.1038/s42003-026-10797-6)
Supplement: Supplementary file 2 [file 42003_2026_10797_MOESM3_ESM.pdf]

Description of Additional Supplementary Files

File name: Supplementary Data 1

Description: Source data underlying all main figure graphs and charts are provided as Supplementary Data.
