# Supplementary material for: A pathological phosphorylation pattern enhances tau cooperativity on microtubules and facilitates tau filament assembly
Source: Commun Biol. 2026 Aug 21;9:1408. doi: 10.1038/s42003-026-10797-6 (PMC13714281; doi:10.1038/s42003-026-10797-6)
Supplement: Supplementary file 4 [file 42003_2026_10797_MOESM2_ESM.docx]

**Supplementary Information**

**A pathological phosphorylation pattern enhances tau cooperativity on microtubules and facilitates tau filament assembly**

Xiangyu Fan^1, 2, 3^, Joey B. Truong^3^, Kyoko Okada^3^, Henry Lin^3^, Kassandra M. Ori-McKenney^3^, Richard J. McKenney^3*^

^1^School of Basic Medical Sciences, Henan University, Kaifeng, Henan, China

^2^Henan Provincial Engineering Centre for Tumor Molecular Medicine, Henan University, Kaifeng, Henan, China

^3^Department of Molecular and Cellular Biology, University of California, Davis, 145 Briggs Hall, Davis, CA, United States

**This PDF file includes:**

Supplementary Table 1

Supplementary Figures 1 to 10

**Supplementary Table 1. Site-specific phosphorylation of eukaryotic expressed 2N4R tau proteins identified by mass spectrometry.**

| site | Degree of phosphorylation | | site | Degree of phosphorylation | |
| --- | --- | --- | --- | --- | --- |
|  | HEK tau | SF9 tau |  | HEK tau | SF9 tau |
| T17 | 0 | (0.06 ± 0.08) % | T231 | (97.47 ± 0.02) % | (99.81 ± 0.28) % |
| Y18 | 0 | (0.01 ± 0.01) % | S235 | (34.46 ± 37.78) % | (29.58 ± 40.68) % |
| Y29 | 0 | (1.10 ± 1.55) % | S237 | (25.96 ± 0.59) % | (49.17 ± 36.31) % |
| T30 | (0.19 ± 0.27) % | (0.41 ± 0.58) % | S238 | 0 | (0.03 ± 0.05) % |
| T39 | 0 | (0.20 ± 0.28) % | S241 | (17.30 ± 24.47) % | (4.80 ± 6.79) % |
| S46 | (10.72 ± 15.16) % | (1.48 ± 1.93) % | T245 | (0.02 ± 0.04) % | (0.01 ± 0.01) % |
| T50 | (3.19 ± 4.51) % | 0 | S258 | (16.84 ± 23.81) % | 0 |
| T52 | 0 | (0.01 ± 0.02) % | S262 | (0.50 ± 0.70) % | 0 |
| S56 | 0 | (0.01 ± 0.02) % | T263 | (0.16 ± 0.22) % | (0.07 ± 0.09) % |
| S61 | 0 | (0.21 ± 0.30) % | S285 | (0.06 ± 0.08) % | (0.003 ± 0.005) % |
| S68 | (1.27 ± 1.64) % | 0 | S289 | (0.01 ± 0.01) % | (0.01 ± 0.02) % |
| T69 | (0.07 ± 0.10) % | 0 | S293 | (0.72 ± 1.03) % | (2.31 ± 3.27) % |
| T71 | (0.29 ± 0.21) % | 0 | S305 | (0.84 ± 1.04) % | (0.56 ± 0.78) % |
| T76 | 0 | (0.25 ± 0.35) % | Y310 | (0.16 ± 0.04) % | (0.39 ± 0.52) % |
| T102 | (5.70 ± 8.05) % | 0 | S316 | (0.30 ± 0.43) % | 0 |
| T111 | 0 | (0.01 ± 0.01) % | T319 | (24.62 ± 34.81) % | 0 |
| S113 | (0.14 ± 0.19) % | (0.02 ± 0.03) % | S320 | (5.65 ± 8.00) % | 0 |
| T123 | 0 | (0.02 ± 0.03) % | S324 | (1.31 ± 0.17) % | (0.54 ± 0.77) % |
| T169 | (4.55 ± 6.43) % | (1.08 ± 1.53) % | S341 | (0.06 ± 0.09) % | 0 |
| T175 | (3.58 ± 4.57) % | (7.78 ± 4.23) % | S352 | (4.53 ± 6.41) % | (44.70 ± 63.22) % |
| T181 | (72.42 ± 11.34) % | (66.42 ± 8.49) % | S356 | (0.51 ± 0.58) % | (0.76 ± 0.76) % |
| S184 | 0 | (0.37 ± 0.53) % | T361 | (0.03 ± 0.04) % | (0.02 ± 0.02) % |
| S185 | 0 | (0.18 ± 0.25) % | T373 | (29.56 ± 41.80) % | 0 |
| S191 | (0.60 ± 0.85) % | (10.80 ± 15.28) % | T377 | (8.13 ± 11.50) % | 0 |
| S195 | (0.97 ± 1.38) % | (2.54 ± 3.59) % | T386 | 0 | (0.08 ± 0.11) % |
| Y197 | (0.35 ± 0.50) % | 0 | Y394 | (0.63 ± 0.89) % | (3.99 ± 5.64) % |
| S198 | (0.25 ± 0.36) % | (22.99 ± 4.81) % | S396 | (11.53 ± 15.69) % | (29.84 ± 32.75) % |
| S199 | (3.45 ± 4.88) % | (4.82 ± 6.82) % | S400 | (1.54 ± 0.13) % | (7.40 ± 9.85) % |
| S202 | (16.81 ± 15.44) % | (47.06 ± 14.81) % | T403 | (18.32 ± 25.69) % | (10.81 ± 15.29) % |
| T205 | (1.42 ± 2.01) % | (0.99 ± 1.41) % | S404 | (22.30 ± 25.04) % | (60.41 ± 17.79) % |
| S208 | (0.07 ± 0. 10) % | (13.76 ± 19.46) % | S409 | 0 | (0.12 ± 0.17) % |
| S210 | (15.87 ± 22.14) % | (42.88 ± 56.19) % | T414 | 0 | (0.12 ± 0.17) % |
| T212 | (0.33 ± 0.47) % | (2.58 ± 2.95) % | S416 | 0 | (1.44 ± 1.69) % |
| S214 | 0 | (0.85 ± 0.87) % | S422 | 0 | (31.71 ± 42.91) % |
| T217 | (7.15 ± 10.08) % | (5.29 ± 1.17) % | T427 | (10.61 ± 14.60) % | (0.83 ± 1.17) % |
| T220 | (0.01 ± 0.01) % | (0.08 ± 0.12) % | S435 | 0 | (0.02 ± 0.03) % |

Average phosphorylation degree:

HEK tau: ~ 9.30 %	SF9 tau: ~ 10.58 %

**Supplementary Figure 1. Size exclusion chromatograph of PreScission Protease treated tau proteins.**

Left: SEC elution profiles of PreScission Protease (PP) treated *E. coli* tau and Sf9 tau. Top Right: SDS-PAGE gel of *E. coli* tau treated with different concentrations of PreScission Protease to remove the N-terminal GFP tag. Bottom right: SDS-PAGE gel of peak fractions shows the subtle elution difference between untagged *E. coli* tau and Sf9 tau.

**Supplementary Figure 2. Lambda Protein Phosphatase treatment restores the single-molecule microtubule binding dynamics and cooperative binding properties of Sf9 tau toward those of non-phosphorylated tau.**

**A.** SDS–PAGE analysis of Lambda Protein Phosphatase (LPP) treated Sf9 GFP-tau. **B.** Kymographs of single Sf9 GFP-tau molecules treated with/without LPP diffusing on microtubules. Scale bars: 10 sec. and 5 µm. **C.** Quantification of the dwell times of Sf9 GFP-tau with/without LPP treatment on microtubules. Cumulative frequency of the dwell time is plotted for each population of tau proteins and fit to a one-phase exponential decay function. The characteristic dwell times derived from the fits (τ) and the corresponding 95% confidence intervals (95% CI, in parentheses) are indicated. Events longer than three pixels were selected and quantified and two independent experiments (n=2) were performed for each condition. Sf9 tau: *N* = 362, R^2^ = 0.996. LPP treated Sf9 tau: *N* = 150, R^2^ = 0.9838. Mean ± SD is shown. **D.** Quantification of landing rates of Sf9 GFP-tau with/without LPP treatment on microtubules. For quantification, microtubules were quantified from two independent experiments (n=2) for each tau protein. Sf9 tau: *N* = 55 microtubules quantified. LPP treated Sf9 tau: *N* = 50 microtubules quantified. *P* values are calculated from an unpaired, two-tailed *t*-test. Mean ± SD is shown. **E.** Quantification of Sf9 GFP-tau with/without LPP treatment binding to microtubules across a range of tau concentrations. Binding curves were fitted to the Hill equation to derive the Hill coefficient and *K_d_*. For each concentration of corresponding tau protein, two independent experiments (n=2) were performed. *N* ≥ 39 microtubules. Mean ± SD is shown. Hill coefficients and *K_d_* are indicated. 95% CI for Hill coefficients: 2.54 to 3.32 (Sf9 tau), 1.80 to 2.12 (LPP treated Sf9 tau). 95% CI for *K_d_*: 51.69 to 55.81 (Sf9 tau), 9.50 to 10.36 (LPP treated Sf9 tau). Insert showing the initial time course of binding. The reported K_d_ and Hill coefficient for each tau variant were derived by fitting a full binding curve from a titration series of tau concentrations.

**Supplementary Figure 3. Uncropped SDS-PAGE for Figure 1.**

**A.** Uncropped SDS-PAGE for Figure 1A. **B.** SDS-PAGE for Figure 1D.

**Supplementary Figure 4. Uncropped SDS-PAGE for Figure 5B.**

**Supplementary Figure 5. Uncropped SDS-PAGE for Figure 5B.**

**Supplementary Figure 6. Uncropped SDS-PAGE for Supplementary Figure 1.**

**Supplementary Figure 7. Uncropped SDS-PAGE for Supplementary Figure 2A.**

**Supplementary Figure 8. SEC chromatograms and corresponding uncropped SDS-PAGE for dGAE and all GFP-Tau proteins purification.**

**Supplementary Figure 9. SEC chromatograms and corresponding uncropped SDS-PAGE for all SNAP-tau proteins purification.**

 **Supplementary Figure 10. Uncropped SDS-PAGE for dGAE concentration determination.**
